# Preparation and characterization of Sorafenib nano-emulsion: impact on pharmacokinetics and toxicity; an in vitro and in vivo study

**DOI:** 10.1007/s13346-024-01530-z

**Published:** 2024-03-02

**Authors:** Dalia Zaafar, Heba M. A. Khalil, Gehad E. Elkhouly, Abanoub Selim Sedeky, Yasmine H. Ahmed, Mona G. Khalil, Yasmin Abo-zeid

**Affiliations:** 1 Department of Pharmacology and Toxicology, Faculty of Pharmacy, Modern University for Technology and Information, Cairo, Egypt; 2 Department of Veterinary Hygiene and Management, Faculty of Veterinary Medicine, Cairo University, Giza, 12211 Egypt; 3 Department of Pharmaceutics and Industrial Pharmacy, Faculty of Pharmacy, Helwan University, Cairo, 11795 Egypt; 4 Helwan Nanotechnology Center, Helwan University, Cairo, 11792 Egypt; 5 Department of Microsystems Engineering (IMTEK), University of Freiburg, Freiburg im Breisgau, Germany; 6 Nanomedicine Lab, Center of Materials Science (CMS), Zewail City of Science and Technology, 6Th of October, 12578 Giza Egypt; 7 Department of Cytology and Histology, Veterinary Medicine Faculty, Cairo University, Giza, 12211 Egypt

**Keywords:** Hepatocellular Carcinoma, Nano-emulsion, Sorafenib, hepG2 Cell line, Pharmacokinetics

## Abstract

Hepatocellular carcinoma (HCC) ranks as the third leading cause of cancer-related deaths worldwide. Current treatment strategies include surgical resection, liver transplantation, liver-directed therapy, and systemic therapy. Sorafenib (Sor) is the first systemic drug authorized by the US Food and Drug Administration (FDA) for HCC treatment. Nevertheless, the conventional oral administration of Sor presents several limitations: poor solubility, low bioavailability, drug resistance development, and off-target tissue accumulation, leading to numerous adverse effects. Nano-emulsion, a nano-delivery system, is a viable carrier for poorly water-soluble drugs. It aims to enhance drug bioavailability, target organ accumulation, and reduce off-target tissue exposure, thus improving therapeutic outcomes while minimizing side effects. This study formulated Sor nano-emulsion (Sor NanoEm) using the homogenization technique. The resultant nano-emulsion was characterized by particle size (121.75 ± 12 nm), polydispersity index (PDI; 0.310), zeta potential (-12.33 ± 1.34 mV), viscosity (34,776 ± 3276 CPs), and pH (4.38 ± 0.3). Transmission Electron Microscopy exhibited spherical nano-droplets with no aggregation signs indicating stability. Furthermore, the encapsulation of Sor within the nano-emulsion sustained its release, potentially reducing the frequency of therapeutic doses. Cytotoxicity assessments on the HepG2 cell line revealed that Sor NanoEm had a significantly (P < 0.05) more potent cytotoxic effect compared to Sor suspension. Subsequent tests highlighted superior pharmacokinetic parameters and reduced dosage requirements of Sor NanoEm in mice. It exhibited an enhanced safety profile, particularly in behavior, brain, and liver, compared to its suspended form. These findings underscore the enhanced pharmacological and toxicological attributes of Sor Nano-emulsion, suggesting its potential utility in HCC treatment.

**Graphical abstract:**

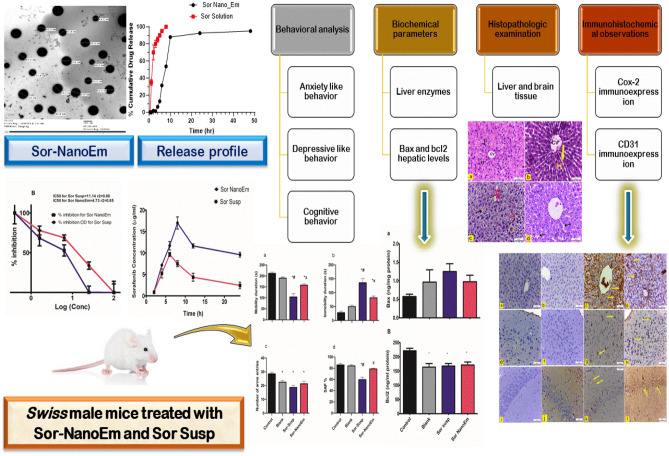

## Introduction

Hepatocellular carcinoma (HCC) stands as the third leading cause of cancer-related mortality globally [1]. Despite advancements in cancer surveillance and diagnosis, many patients still have late-stage diagnoses [2–4]. Various treatment strategies for HCC, including surgical resection, liver transplantation, liver-directed therapy, and systemic therapy, have been employed [5]. Sorafenib (Sor) emerged as the pioneering systemic drug sanctioned by the US Food and Drug Administration (FDA) for HCC treatment [6, 7]. Functioning as a multi-kinase inhibitor, Sor inhibits serine-threonine kinases Raf-1 and B-Raf, along with the receptor tyrosine kinase activity of vascular endothelial growth factor VEGFRs and platelet-derived growth factor receptor (PDGFR), pivotal elements in the molecular pathogenesis of HCC [6]. Additionally, atezolizumab plus bevacizumab garnered FDA approval as a first-line treatment for HCC, exhibiting superior survival outcomes compared to Sor [8]. However, the higher treatment costs associated with these drugs, in contrast to Sor therapy, positioned Sor as a key therapeutic agent for HCC treatment [9, 10].

Despite its efficacy, Sorafenib (Sor) faces several limitations compromising its effectiveness in cancer therapy, including poor solubility, low bioavailability, drug resistance development, and non-selective action targeting healthy and tumor tissues [10–12]. The latter contributes to observed side effects during Sor's clinical application, potentially impacting the patient's quality of life [8]. These side effects may include elevated blood pressure, proteinuria, hand-foot skin reactions, asthenia, anorexia, diarrhea, and weight loss. Moreover, Sorafenib treatment is often associated with grade 3–4 drug-related adverse events in approximately 50% of patients, leading to withdrawal rates of around 15% [3, 13]. Thus, identifying a delivery system capable of enhancing drug solubility bioavailability, overcoming resistance, and selectively targeting tumor tissues becomes crucial. Such an improvement could bolster its pharmacological activity while reducing side effects, ultimately enhancing the therapeutic management of HCC.

Nanotechnology, an innovative field utilized for decades, has significantly advanced the efficacy of anticancer treatments [14]. Nano-delivery systems have been applied for selective delivery of drugs to diseased organs, minimizing off-target accumulation, this is mainly attributed to the enhanced permeability and retention effect (EPR), a pathophysiological condition characterized the tumor, where tumor vasculature is leaky allowing the accumulation of nano-delivery systems sized less than 200 nm specifically at the tumor site and overcome their accumulation at organs with normal vasculature (non-leaky) [15, 16]. This could be associated with reducing drug frequency [17], and subsequently reducing drug side effects [16–18].

These systems were also reported to overcome drug resistance, enhance pharmacokinetic parameters, improve the bioavailability of poorly soluble drugs, regulate drug release, and enhance drug stability [19–23]. Leveraging nano-delivery systems to transport Sor into the liver actively holds promise for improving its therapeutic efficacy while diminishing its potential side effects.

Several attempts have been made to develop diverse nano-formulations of Sor, including lipid polymer hybrid nanoparticles [24], liposomes [25], self-emulsifying drug delivery systems [26], cyclodextrin-modified silicon nanoparticles [27], nanogels [28], diatomite nanoparticles [29], nano colloidal carrier [30], and pullulan nanoparticles [31]. However, most of these studies focused on non-oral applications [32].

The present study aimed to encapsulate Sor into a nano-emulsion for oral delivery. Nano-emulsions, typically sized between 20 and 200 nm and composed of oil and aqueous phases [28, 33], made it an ideal system for dissolving large amounts of lipophilic drugs. Additionally, they enhance drug stability by reducing enzymatic degradation and clearance [34]. This research focused on encapsulating Sor into a nano-emulsion to demonstrate that a nano-delivery system applied orally might potentially enhance the therapeutic efficacy of Sor and diminish its side effects compared to conventional therapy.

To ascertain our hypothesis, the anticancer efficacy of Sor nano-emulsion (Sor NanoEm) was assessed in vitro on HepG2 cells and compared to Sorafenib suspension (Sor susp), representing the conventional therapy. Subsequently, the study aimed to determine the lethal dose 50 (LD 50) for Sor NanoEm and Sor susp, both in vitro and in vivo using male Swiss mice. After investigating the safety of Sor NanoEm compared to conventional therapy, the pharmacokinetic parameters for Sor susp (100 mg/kg) versus Sor NanoEm (30 mg/kg) were tracked after a single oral dose.

Furthermore, an in vivo study examined animal behavior for 21 consecutive days following a single oral dose to assess the potential side effects of each formulation. This was complemented by histopathological examination and biochemical analysis.

## Materials and methods

### Materials

#### Drugs and chemicals

Sorafenib, generously provided by Professor Tamer Nasr (Nexavar®, Bayer, USA), was utilized in this study. The pure Sorafenib powder was obtained by grinding the tablets and dissolving them in acetonitrile/water (90/10, v/v), as previously described [35]. Following the filtration to remove drug excipients and additives, the resulting solution underwent evaporation using a rotary evaporator to yield the pure Sorafenib powder employed in the present studies.

Tris, glycine, SDS, Tween 80, Polyethylene glycol 600 (PEG 600), Dimethyl sulfoxide (DMSO), phosphate buffer saline (PBS) tablets, and Castor oil were procured from Sigma Aldrich. All chemicals were of analytical grade. The HepG2 liver cancer cell line was purchased from the American Type Culture Collection (HB-8065).

### Methodology

#### Preparation and characterization of Sor NanoEm

Sor NanoEm was formulated using castor oil, phosphate-buffered saline (PBS, 10 mM, pH 7.4), Tween 80, and PEG 600 at 10:5:2.5:2.5, respectively. Initially, Sorafenib powder (40 mg) was dissolved in PEG 600 (as a co-surfactant) and Tween 80 (as a surfactant). Subsequently, castor oil was added, and the mixture was ultrasonicated in a water bath sonicator (Elmasonic S 30 H, Germany) for 5 min. Following this, the blend was placed in a cold-water bath, and a solution of PBS (10 mM, pH 7.4) was gradually added dropwise under homogenization (Daihan Homogenizer, ZS version, Korea) at 750 rpm for 11 min. The formed nano-emulsion contained Sor at a concentration 2 mg/ml was used for characterization and other experimental work.

The Sor NanoEm droplet size, PDI, and zeta potential were assessed using a Malvern Zeta-sizer Nano ZS (Malvern Instruments Ltd, Malvern, UK) at a constant temperature of 25 °C ± 0.1. Samples were diluted in PBS (10 mM, pH 7.4) to maintain a count rate ranging from 50 to 300 KCPs. For morphological analysis, Transmission Electron Microscopy (TEM) (H-700, Hitachi Ltd., Japan) was employed where samples were diluted (1:50) with PBS (10 mM, pH 7.4). A drop of the diluted solution was applied to a mesh copper grid coated with carbon film. After drying for 5 min, a drop of phospho-tungstic acid (2% w/w) was added to the grid for 50 s, followed by removal of excess liquid using filter paper.

Additionally, the pH and viscosity of the nano-emulsion were measured at room temperature. pH was determined using an Ohaus Economical pH bench meter (Starter 3100, USA), calibrated with three standard buffer solutions (pH 4, 7, and 10). Viscosity was assessed using Lamy Rheology (B-one Plus, Germany) with spindle 6 at 50 rpm for 3 min.

#### UV–Visible spectrophotometric analysis and its validation

The UV–Visible Spectrophotometric analysis of Sor was validated, following our established protocol (Abo-zeid et al. 2022), with the following modifications. The linearity of Sor at pH 1.2 and pH 7.4 was determined over concentrations ranging from 0.04 to 2.5 µg/ml and 0.3 to 10 µg/ml, respectively, where Sor was dissolved in DMSO: PBS (10 mM) at a molar ratio of 1:2, followed by sample analysis at λmax 264 nm.

The accuracy and precision of inter-day and intra-day variations were evaluated using Sor concentrations of 0.31, 1.5, and 2.5 µg/mL at pH 1.2, and 2, 2.5, and 10 µg/ml at pH 7.4. Each concentration was analyzed three times on the same day at different intervals (intra-day variation) or on separate days (inter-day variation). Accuracy and precision were computed using Eqs. (1) and (2), respectively.

1 $$\mathrm{Accuracy }= (\mathrm{M }-\mathrm{ N}) / ({\text{N}}) * 100$$

M is the mean value of Sor concentration measurements, while N is the theoretical concentration.

2 $$\mathrm{Precision }(\mathrm{\% RSD}) =\mathrm{ SD }/\mathrm{M }* 100$$

RSD is the relative standard deviation, SD is the standard deviation of measurements, and M is the mean value of Sor measurements.

#### Release study

Sor NanoEm and Sor solution (Sor pure powder dissolved in DMSO) were investigated for drug release using a modified version of the protocol outlined in previous studies [30, 32, 36]. In this study, NanoEm or Sor DMSO solution (1 ml, 2 mg/ml) was placed within a dialysis membrane bag (Dimension; 5 cm × 2.5 cm, pore size 2.4 nm, molecular weight cutoff 12–14 kDa) that had been pre-conditioned with the release medium [DMSO: PBS (10 mM, pH 1.2) (1:2)] for 24 h. The release experiment involved an initial 2-h duration simulating gastric pH, followed by relocation of the dialysis membrane to another release medium [DMSO: PBS (10 mM, pH 7.4) (1:2)] to simulate intestinal pH. Drug release was monitored for up to 48 h under continuous stirring (100 rpm) at 37°C. Samples (3 mL) were periodically withdrawn and replaced with an equal volume of fresh-release medium at specific intervals. Each time-point was run in triplicate and analyzed by UV–Visible spectrophotometry at λmax 264 nm.

#### Release kinetics

The kinetics of Sor release from the nano-emulsion was assessed using DD Solver software, following the methodology reported in earlier studies [34, 36]. The release data underwent fitting into various release kinetic models, and the model that exhibited the highest coefficient value of R2 was chosen as the most suitable mathematical representation for the kinetic release profile.

#### Cell growth inhibition and cytotoxic effect of Sor-Susp and Sor-NanoEm using MTT assay on HepG2 cell line

##### Cell line culture

Cells were cultured in DMEM (Invitrogen/Life Technologies) supplemented with 1% penicillin–streptomycin and maintained in a humidified atmosphere with 5% CO_2_ at 37°C. The experiments were replicated in three independent trials.

##### MTT cytotoxicity assay

The MTT assay involved the determination of the percentage of cell viability after incubation with tested samples, followed by calculating the IC50 value for each sample to compare their efficacy in inhibiting HepG2 cells, following established protocols [35, 37].

The effect of Sor Susp and Sor NanoEm on cell proliferation was evaluated using the MTT (Cat. no. 298–93-1; Cyman Chemical Co., USA) uptake method. Around 3 × 10^3^ cells were seeded per well in a 96-well plate and incubated for 12 h. The following day, cells were exposed to varying Sor Susp and Sor NanoEm concentrations at 37°C for 48 h. Subsequently, MTT reagent (5 mg/ml; Sigma Aldrich) was added to each well and incubated at 37°C for 4 h. The absorbance was measured at 450 nm using a ROBONIK P2000 Elisa Reader (Thermo Fisher Scientific, Inc.).

### In-vivo mice experimental studies

#### Animal housing and Statement of ethics

The experimental procedures adhered to the NIH Guidelines for the Care and Use of Laboratory Animals and were approved by the Institutional Animal Care and Use Committee at the Faculty of Veterinary Medicine, Cairo University (Approval number: Vet CU 09092023768).

All procedures were conducted following the ARRIVE guidelines to ensure the utmost care for the animals used in the research [38].

Male Swiss mice weighing 25–27 g were procured from a commercial supplier (National Cancer Institute, Cairo, Egypt) for all in-vivo experiments, including determining lethal dose 50, performing pharmacokinetic study, and determining the main biological parameter level. Mice were acclimatized for one week before the commencement of the experiments and housed in plastic shoebox-type cages (5 mice/ cage, 27 × 13 × 13 cm) provided with coarse saw dust as a bedding material. They were maintained under standard laboratory conditions (temperature: 25˚C; humidity: 50%; normal light/dark cycle) and had ad libitum access to a well-balanced commercial diet and water throughout the study.

#### Lethal dose 50 determination

In this study, we initially determined the LD50 of the Sor-NanoEm formulation. Mice underwent a 12-h fasting period before receiving a single oral administration of Sor-NanoEm.

##### Estimation of the dose range and percentage of mortalities

The experiment started with a gavage administration of a single oral dose of Sor-NanoEm (150 mg/kg) to mice the following day after a prior evening fast. Mice were individually placed with access to food and water for the subsequent two hours. To establish the LD50, a modified 4-level Up and Down Procedure was employed, as previously reported [39].

This involved an increase in the number of mice at each subsequent level: three mice at the first level, five at the second, seven at the third, and so forth. If over 50% of the mice died, the subsequent level dose was reduced. Conversely, if fewer than 50% of the mice succumbed, the dose was increased. Signs were meticulously recorded at each dosage level throughout the experiment.

#### Pharmacokinetics study

The mice were divided into two groups, each consisting of 15 animals. Group I received Sor-NanoEm at a concentration of 30 mg/kg. In comparison, Group II was administered free Sor powder suspended in 2.5% carboxymethyl cellulose (CMC) at a dose of 100 mg/kg via oral gavage. Blood samples (200 µL) were collected from the retro-orbital plexus of the mice using heparin-containing tubes at specified time intervals. Plasma proteins were precipitated from the blood samples by centrifugation at 12,000 rpm for 10 min. Subsequently, Sor levels in the extracted plasma samples were estimated using the following HPLC method.

#### High-performance liquid chromatography (HPLC) assay

The concentrations of Sor were measured using high-performance liquid chromatography (HPLC) techniques equipped with ultraviolet (UV) detection (HPLC system Echromatecg Mod. C 1620 liquid chromatography).

A reversed-phase C18 column facilitated the gradient elution of the mobile phase from water (eluent A) to acetonitrile (eluent B) at a flow rate of 1.0 mL/min. The gradient transitioned linearly from 60% eluent A and 40% eluent B to 17.5% eluent A and 82.5% eluent B (Brocks et al., 2010). Subsequently, all measured parameters underwent statistical analysis.

#### Evaluation of Pharmacokinetic Parameters

The R® software version 3.5.2 was employed to calculate various pharmacokinetic parameters, including the area under the plasma concentration–time curve from zero to the time of the last measurable concentration (AUC0-t), the area under the plasma concentration–time curve from zero to infinity (AUC0-∞), maximum plasma concentration (Cmax), elimination rate constant (Kel), half-life in the elimination phase (t_1/2_), apparent plasma drug clearance (Cl), and apparent volume of distribution (Vd).

#### In-vivo investigation of the impact of Sor-NanoEm on mice's behavioral and biochemical parameters

##### Study design

Twenty-four male albino mice were randomly distributed into four groups (n = 6) as follows:

- Group I: Control—Mice received orally administered 2.5% CMC solution (2 ml/kg/day), the vehicle used to suspend Sor to formulate Sor Suspension.
- Group II: Blank—Mice were orally administered Blank nano-emulsion (2 ml/kg/day), (nano-emulsion prepared as described in section 'Preparation and characterization of Sor NanoEm' but in the absence of Sor).
- Group III: Sor-Susp group—Mice were orally administered Sor suspended in 2.5% CMC (100 mg/kg daily) [13, 40].
- Group IV: Sor-NanoEm group—Mice were orally administered a daily dose of Sor-NanoEm formulation (30 mg/kg daily).

**Fig. 1:**
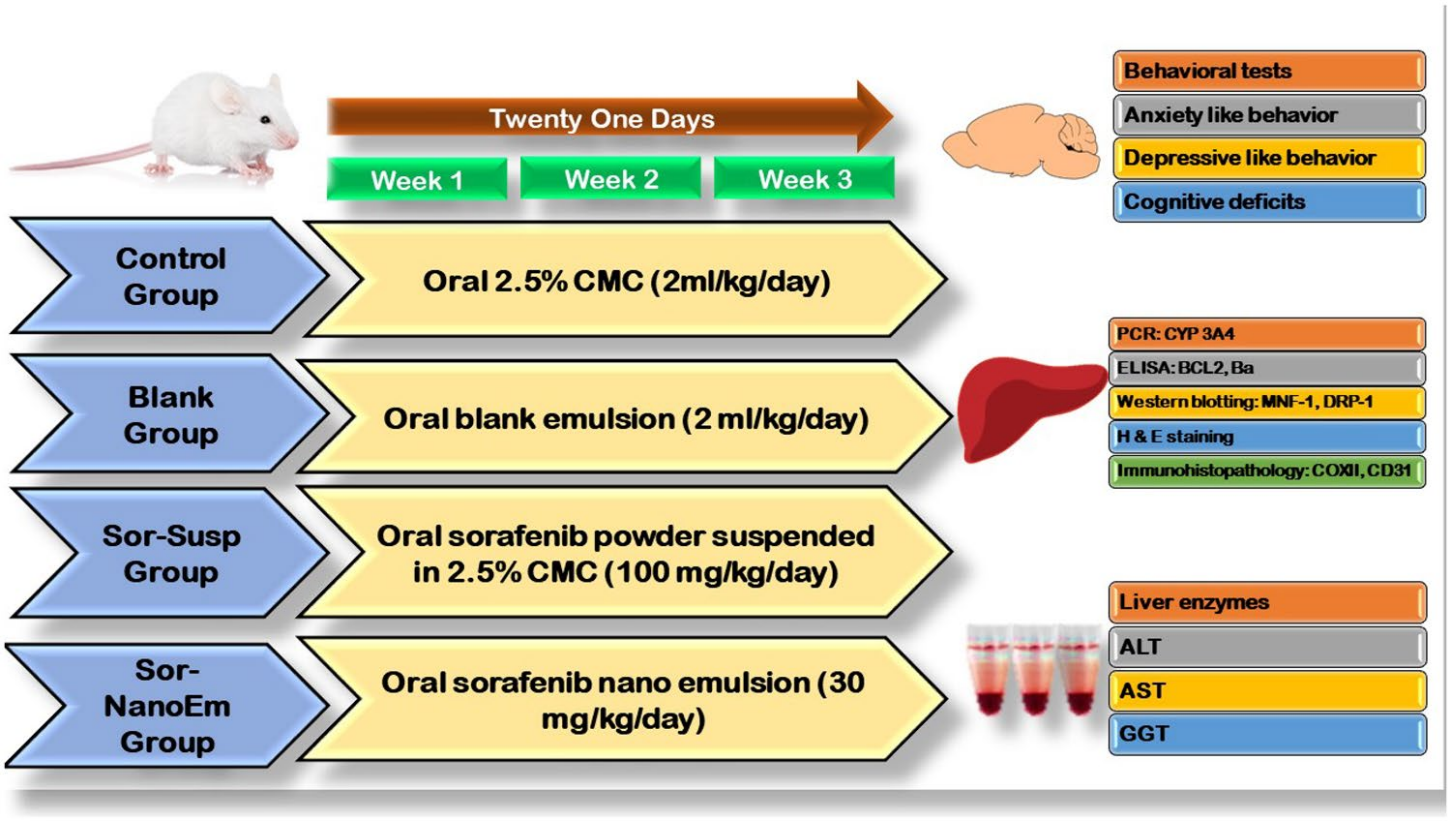
A flow chart showing the experimental design of the in vivo study

The treatment for each group continued for 21 consecutive days, as illustrated in Fig. 1.

#### Behavioral study

After completing the final dose of treatment, all mice were placed in the behavioral analysis room and allowed to acclimate to the presence of the experimenter for 2 h. Subsequently, the mice underwent assessments to evaluate sickness-like behaviors, encompassing anxiety, depression, and cognitive deficits through distinct behavioral tests:

#### Anxiety-like behavior using open field test and dark–light activity box

Mice underwent an open-field test, a standard method for assessing rodent locomotion and exploratory behavior [41]. Each mouse was placed in one corner of a wooden box divided into 16 squares, and its activity was recorded for 3 min. Measured parameters included the number of squares crossed and rearing frequency [42]. Additionally, a dark–light activity box test was conducted, where mice spent 5 min in a box with two chambers: one dark and one illuminated. The parameters assessed were the frequency and duration of entries into light or dark chambers [43].

#### Depressive-like behavior using forced swim test

The forced swim test is a screening tool for assessing the antidepressant activity of drugs [44]. Mice were individually tested for 6 min in a warm, water-filled cylindrical container. The initial 2 min of the video recording were excluded, and the subsequent 4 min were assessed for mobility and immobility duration. Mobility behaviors such as swimming, diving, and climbing against the bottle wall were considered, while immobility referred to minimal movements to keep the mouse's head above the water. After the test, each mouse was dried in a wooden chip-filled cage before returning to its original cage. Fecal pellets were removed between each mouse, and water changes were made if necessary [45].

#### Cognitive deficits using the Y-maze test

The Y-maze test assesses spatial working memory, reliant on hippocampal integrity. Each mouse was placed in a Y-shaped wooden maze and tested for 5 min. Parameters measured included the number of arm entries and the spontaneous alternation percentage (SAP). SAP was computed using the formula [40, 42]:

$$\mathrm{SAP }=\mathrm{ number of alternations}/(\mathrm{total arm entries }- 2) \times 100$$

#### Euthanasia, blood, and tissue sampling

After the conclusion of the behavioral tests, mice were euthanized using an intraperitoneal injection of a ketamine (100 mg/kg, i.p.) and xylazine (10 mg/kg, i.p.) mixture [46]. Blood samples were collected from the inner canthus of each mouse's eye and centrifuged at 3000 rpm for 15 min to obtain separated sera, which were stored at -80°C until liver function parameters were evaluated. Subsequently, the mice were euthanized by cervical dislocation. The liver and brain were harvested and rinsed with cold saline. Half of each organ was preserved at -80°C for biochemical analysis, while the remaining halves were fixed in 10% formalin-buffered saline for histopathological and immunohistochemical examinations.

Tissue homogenates of the brain and liver were prepared in phosphate buffer (0.05 M, pH 7) using a polytron homogenizer at 4°C. The homogenate underwent centrifugation at 10,000 rpm for 20 min to eliminate cell debris, unbroken cells, nuclei, erythrocytes, and mitochondria. The resulting supernatant (cytoplasmic extract) was utilized for subsequent biochemical analysis following the manufacturer's guidelines.

Fixed tissue samples were dehydrated, treated with xylene, and embedded in paraffin. Sections of 3μm thickness were generated, deparaffinized, and stained with hematoxylin and eosin (H&E) for histological examination.

#### Biochemical analysis

##### Evaluation of different liver enzymes levels

Serum levels of various liver enzymes—alanine aminotransferase (ALT), aspartate aminotransferase (AST), Gamma Glutamyl Transferase 1 (GGT)—as well as levels of Cytochrome P450 3A4 (CYP3A4) in liver tissue were determined using the ELISA sandwich technique following the protocols provided by BIOMATIK (Ontario, Canada) for ALT (Catalog number #EKU02211) and AST (Catalog number #EKE62019), Elabscience (USA) for GGT (Catalog number #E-EL-R0404), and LifeSpan BioSciences, Inc. (Washington, USA) for CYP 3A4 (Catalog number #LS-F27379). The concentration of each enzyme was evaluated by measuring the optical density using an automated ELISA reader set at 450 nm.

##### Estimation of hepatic tissue levels of Bcl2 and Bax using the ELISA technique

Sandwich enzyme-linked immunosorbent assay (ELISA) kits from Cloud-Clone Corp were employed to assess the levels of B-cell leukemia/lymphoma 2 (Bcl2) and Bcl2 associated X protein (Bax) in liver tissue homogenate, following the manufacturer's instructions (Catalog number #SEA778Ra for Bcl2 and #SEB343Ra for Bax), (Cloud-Clone Corp. (Wuhan, China).

##### Histopathological examination

The liver, cerebrum, and hippocampus specimens were collected and fixed in 10% neutral buffered formalin for 48 h. Subsequently, they were dehydrated using increasing concentrations of ethyl alcohol, cleared with xylene, and embedded in paraffin wax. Sections of 4 µm thickness were prepared, deparaffinized, and stained with hematoxylin and eosin (H&E) for examination under a light microscope [47].

#### Immunohistochemical examination

##### Cyclooxygenase 2 protein (COX-2) immunostaining

Cyclooxygenase 2 (COX-2) (Catalog # PA1-37,505, Thermos Fisher Scientific, USA) was employed in the present study to identify liver, cerebral cortex, and hippocampus inflammation. The method used was outlined according to Côté and colleagues [48].

##### Cluster of differentiation 31 (CD31) immunostaining

Cluster of differentiation 31 (CD31) (Catalog # 14–0311-82, Thermos Fisher Scientific, USA) was used in the current study to identify vascular lesions in the liver and cerebrum. The method used was outlined according to Hsu and colleagues [49].

##### Image analysis

The assessment of immunohistochemical observations (area percentage) involved analyzing sections stained with anti-COX-2 and anti-CD31 using a digital Leica Quin 500 image analysis system (Leica Microsystems, Switzerland) located at the Faculty of Dentistry, Cairo University. Statistical analysis was conducted on each specimen's mean values and standard deviations.

#### Statistical analysis

The data analysis was performed using GraphPad Prism 9.0 software. Mean values and standard deviations were utilized to represent the experimental findings. IC50 values were calculated using sigmoid nonlinear regression [50]. For data analysis, one-way variance analysis (ANOVA) and the post hoc Tukey test for multiple comparisons were employed. Statistical significance was set at a P-value below 0.05.

## Results

### Preparation and characterization of Sor-NanoEm

The formed Sor-NanoEm were prepared and characterized as described in section 'Drugs and chemicals', and the data obtained was presented in Table 1. Droplet size was shown as the average value of droplet size diameter (nm) ± standard deviation (SD), and it was 121.75 ± 12 nm that was significantly (P < 0.05) larger than the size recorded for blank Nano-Em, 80.33 ± 29.78 nm. The PDI values for the formed Sor Nano-Em indicated a monodisperse sample where the PDI value was ≤ 0.3, and it was close to the PDI value of Blank Nano-Em (0.330).

**Table 1:** Physicochemical characterization of Sor NanoEm and blank Nano-Em

Sample name	Droplet Size (D nm ± SD)	Zeta Potential (mv ± SD)	PDI	pH	Viscosity (Cps)
Sor-NanoEm	121.75 ± 12	- 12.33 ± 1.34	0.310	4.38 ± 0.3	34,776 ± 3276
Blank	80.33 ± 29.78	- 11.37 ± 2.84	0.330	4.68 ± 0.2	30,111 ± 4197

The values of zeta potential recorded for Nano-Em were presented as zeta potential (mv) ± SD, and it was—12.33 ± 1.34mv for Sor NanoEm, was close to the zeta potential value of blank Nano-Em,—11.37 ± 2.84 mv. The low negativity values might be attributed to using a non-ionic surfactant, Tween 80, as an emulsifying agent [51].

The viscosity recorded for Sor-NanoEm was 34,776 ± 3276 Cps, and it was non-significantly (P > 0.05) higher than the viscosity recorded for blank Nano-Em, 30,111 ± 4197. pH recorded for Sor-NanoEm and Blank Nano-Em was 4.38 ± 0.3 and 4.68 ± 0.2, respectively.

**Fig. 2:**
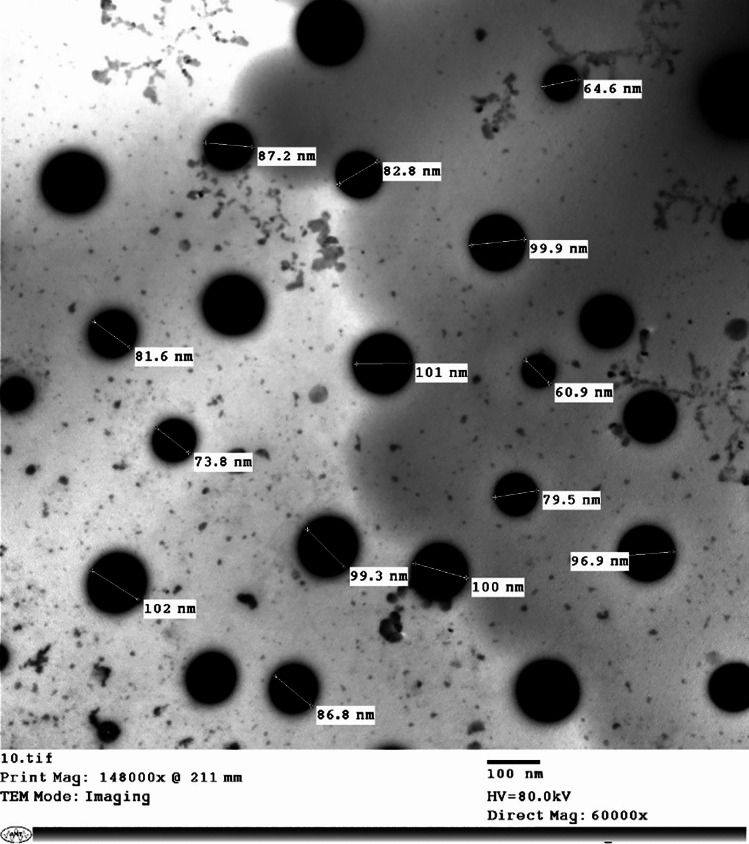
TEM image of Sor-NanoEm, scale bar 100 nm

Transmission electron microscopy images of Sor-NanoEm were presented in Fig. 2. Images showed spherical Nano-Em with no sign of aggregation, and the droplet size ranged from 60.9 to 102 nm.

### UV–Visible Spectrophotometric analysis and its validation

The validation of UV–visible spectrophotometric analysis of Sor was performed, as described in section 'UV–Visible spectrophotometric analysis and its validation'. The linearity of Sor at pH 1.2 and 7.4 was demonstrated over a concentration range from 2.4 ng to 2.5 µg and 0.3 to 10 µg/mL, and the correlation coefficient (R2) values were 0.9931 and 0.9978, respectively. The values of the limit of detection (LOD) and limit of quantification (LOQ) at pH 1.2 were 0.279 and 0.846, while their values at pH 7.4 were 1 and 3 μg/mL, respectively.

The intra-day and inter-day variations at pH 1.2 were performed using three concentrations (0.321, 1.5, and 2.5 μg/mL). The accuracy for intra-day and inter-day variations ranged from 0.36 to 15.39 and 0.88 to 52.48, respectively. The precision (%RSD) was also calculated, ranging from 0.02% to 0.2% and 2.5% to 20.12% for intra-day and inter-day variations, respectively. For analysis at pH 7.4, the accuracy determined for intra-day and inter-day variations was 1.78 to 11.79 and from 1.94 to 11.59, respectively. The precision values determined for Sor analysis at pH 7.4 were 0.02 to 0.17 and from 2.5 to 20.17 for intra-day and inter-day variations, respectively.

### Release study

**Fig. 3:**
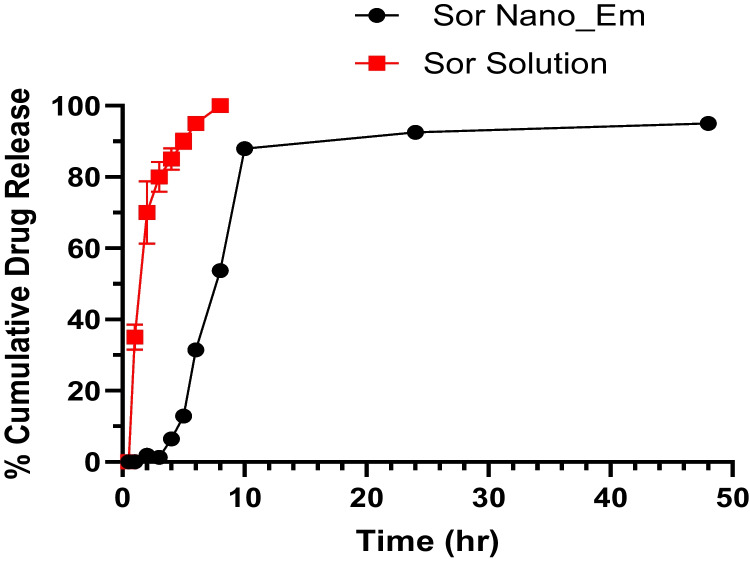
In vitro release profile of Sor suspension (Sor-Susp) and Sor-NanoEm over 48 h where release medium was DMSO: PBS (10mM, pH 1.2) for the first 2 h, then after, release study was continued in DMSO: PBS (10mM, pH7.4) at 37°C ± 0.5°C

A release study for Sor suspension and Sor-NanoEm was performed as described in section 'Preparation and characterization of Sor NanoEm'. The dialysis bag diffusion technique presented release graphs in Fig. 3. As discussed, the cumulative Sor release percentage was determined by UV–visible spectrophotometric analysis. As revealed in Fig. 3, the cumulative release percentage after 8h for Sor solution was 100%, consistent with the literature. Contrary to Sor Nano-Em, drug release percentage at 8 and 10h was 53% and 87.93%, respectively. The release of Sor was plateaued after 10 h for Sor-NanoEm, reflecting the sustained release properties from Sor Nano-Em.

Release kinetic models were applied to assess the mechanism of Sor release from Sor-NanoEm. R2 values were obtained by linear regression analysis, and they were the best-fitting zero-order kinetics for Sor-NanoEm.

### Cell growth inhibition and Cytotoxic effect of Sor-Susp and Sor-NanoEm using MTT assay on HepG2 Cell line

**Fig. 4:**
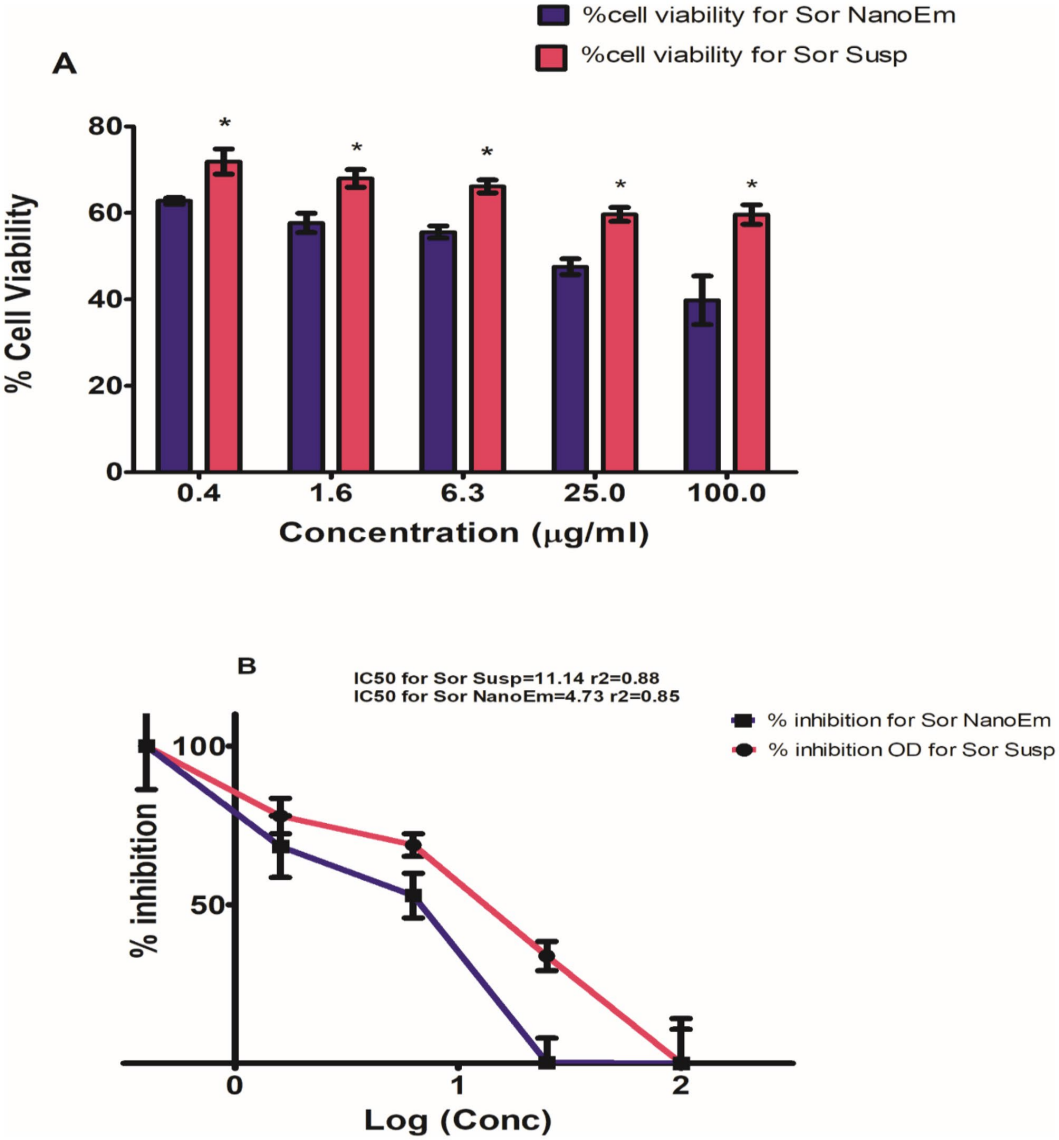
MTT cytotoxicity study of Sor Nano-Em on HepG2 liver cancer cell line; **A** Cell viability percentage of HepG2 after treatment with sorafenib suspension (Sor Susp) and Sor NanoEM. Significant differences in the cytotoxic effect on HepG2 cells are denoted with asterisks * at P < 0.05. **B** Determination of cytotoxic concentration (µg/ml) responsible for the death of 50% of HepG2 cells (IC50) for Sor Susp and Sor NanoEm formulas

The viability percentage of HepG2 cells treated with Sor suspension and its Nano-Em forms is depicted in Fig. 4A. The cell viability percentage exhibited dose-dependency, with an increase in Sor concentration correlating with a decrease in cell viability percentage. However, a significantly (P < 0.05) more significant reduction in cell viability percentage was observed with Sor NanoEm compared to Sor suspension.

To determine the enhanced cytotoxic effect of Sor Nano-Em over Sor suspension, IC50 values were calculated for both formulations using the dose–response curve presented in Fig. 4B. The results indicated an IC50 value of 4.73 µg/ml (r2 = 0.85) for Sor NanoEm and 11.14 µg/ml (r2 = 0.88) for Sor suspension. These findings highlight the superior cytotoxic activity of Sor Nano-Em against HepG2 cells compared to Sor suspension.

### Toxic lethal dose 50 determination

All mice administered 150 mg/kg of Sor-NanoEm via oral gavage died within one hour of the experiment. Subsequently, a 75 mg/kg dose was tested, resulting in the death of three out of four mice. Following this, a 50 mg/kg dose was administered, leading to the death of a single mouse. Finally, 30 mg/kg was given, with all animals surviving.

Severe diarrhea accompanied by shivering was promptly observed after the administration of toxic doses (ranging from 50 to 150 mg/kg). Additionally, convulsions became increasingly visible and hazardous over time. When the 30 mg/kg dose was administered, No Observed Adverse Effect Level (NOAEL) was reached, as all mice survived.

**Fig. 5:**
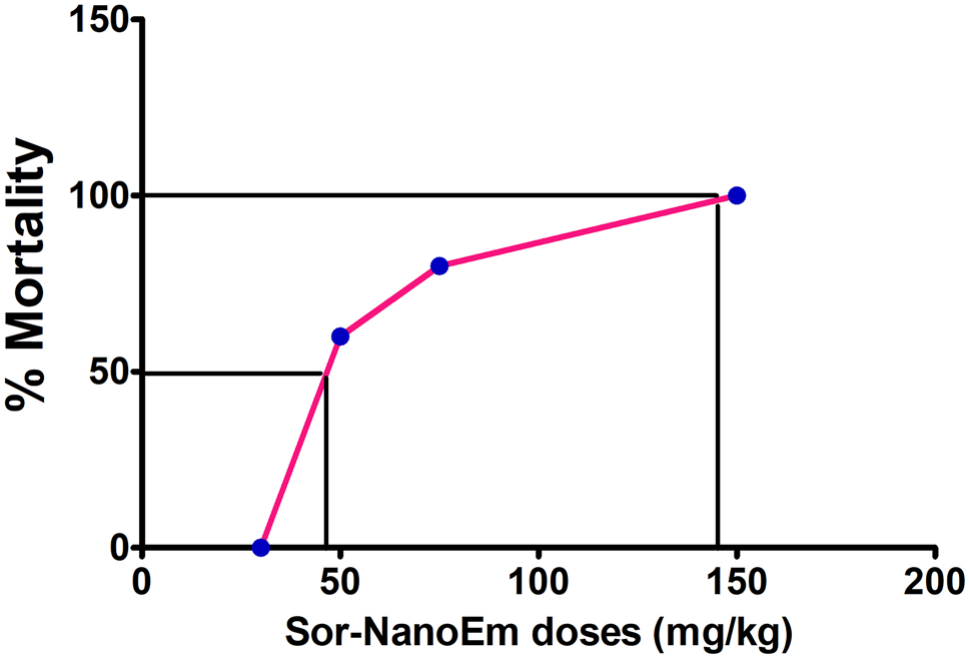
Dose–response mortality curve of oral Sor Nano-Em in mice. Percentage lethality values are plotted against the concentration of the formula. The LD50 value is indicated

Figure 5 illustrates these observations on a scale depicting the percentage of mouse mortality versus the administered dose. Based on this data, a nonlinear regression fitting procedure was utilized to estimate the oral LD50 and LD100 to be 48 mg/kg and 150 mg/kg, respectively.

The onset of toxic signs and symptoms following oral administration of Sor NanoEm displayed dose dependency. At a dose of 150 mg/kg, poisonous symptoms appeared two hours post-administration, primarily involving diarrhea and symptoms related to CNS stimulation. However, at doses of 75 and 50 mg/kg, toxicity signs emerged between 6 to 12 h after drug administration, characterized by trembling behaviors such as chewing, licking, rolling, arching, shivering, and coarse whole-body tremors. Subsequently, animals displayed signs of distress, such as gasping and labored breathing, ultimately leading to mortality.

Conversely, no observable signs of toxicity were evident after oral administration of 30 mg/kg of Sor NanoEm. No adverse effects on the CNS were observed at this dosage, and all animals remained alive.

### Pharmacokinetics Study

**Fig. 6:**
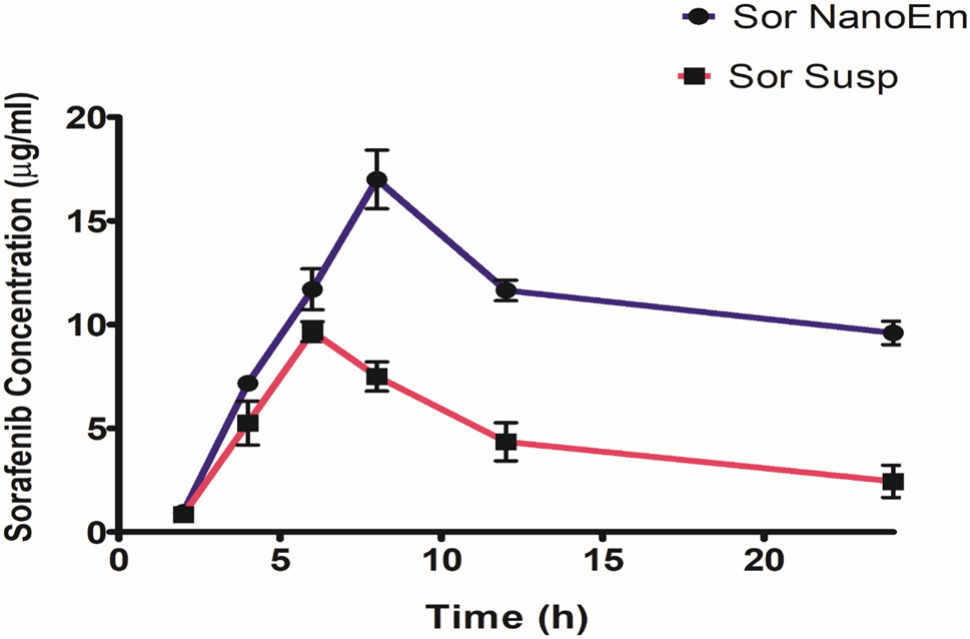
The sorafenib plasma concentration–time profiles in mice receiving sorafenib suspension (Sor-Susp) (100 mg/kg) and Sor NanoEm (30 mg/kg). Data were presented as mean ± standard deviation

The plasma concentration–time curve after administering a single oral dose of Sor susp (100 mg/kg) and Sor NanoEm (30 mg/kg) was depicted in Fig. 6. Corresponding pharmacokinetic parameters for each Sor formulation were presented in Table 2. The data obtained revealed a significant (P < 0.05) increase in the drug's Cmax, AUC0-t, AUC0-∞, and half-life but demonstrated a reduction of Kel, Vd, and CL for Sor NanoEm compared to Sor susp.

**Table 2:** Plasma pharmacokinetic parameters of sorafenib suspension (100 mg/kg) and Sor Nano-Em (30 mg/kg) after the administration of a single oral dose

Pharmacokinetic Parameter	Sor NanoEm	Sor Susp
C max (µg/mL)	17.33 ± 0.24	9.6 ± 0.1
AUC0-t (µg.h/mL)	88.9 ± 0.16	58.4 ± 0.21
AUC0-∞(µg.h/mL)	232.2 ± 0.21	23.27 ± 0.145
Kel (h^−1^)	0.0135 ± 0.0008	0.051 ± 0.0011
T _½_ (h)	50.93 ± 0.174	12.96 ± 0.177
Vd (mL)	7.2 ± 0.17	24.02 ± 0.186
CL (L/h)	0.112 ± 0.0095	1.111 ± 0.07

AUC0-t, area under the plasma concentration–time curve from zero to the time of last measurable concentration; AUC0–∞, area under the plasma concentration–time curve from zero to infinity; Cmax, maximum plasma concentration; Kel, elimination rate constant; t1/2, half-life in the elimination phase; Cl, apparent plasma drug clearance; Vd, apparent volume of distribution. Data were presented as mean ± SD, bold font represented significant difference when P is less than 0.05

### Behavioral analysis

#### Anxiety like behavior

**Fig. 7:**
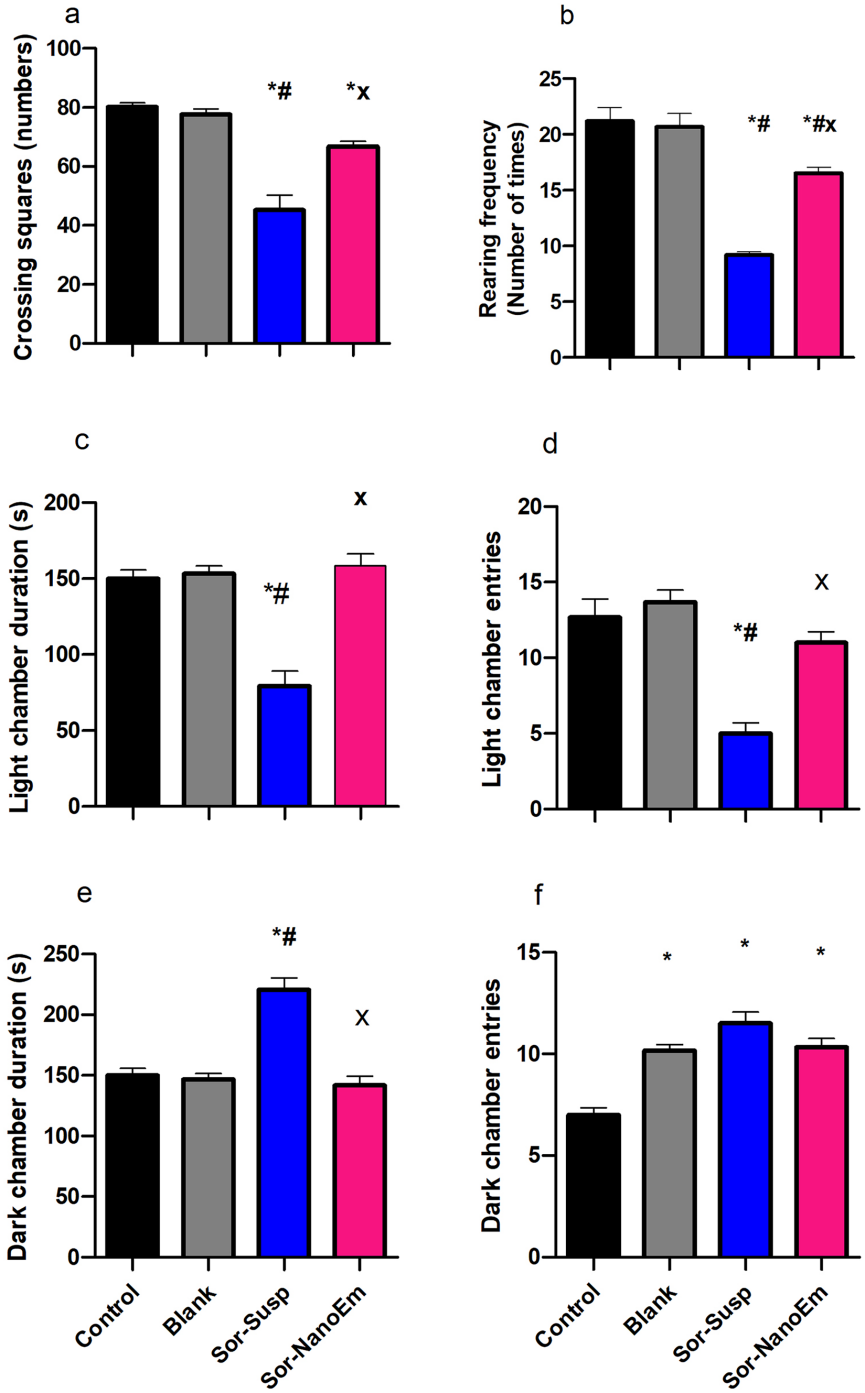
Effect of Sora- Susp and Sor Nano-Em administration on the anxiety-like behavior of the mice. **a** Open field test (number of crossing squares), **b** Open field test (Rearing frequency), **c** Light dark activity box (light chamber duration), **d** Light dark activity box (light chamber entries), **e** Light dark activity box (dark chamber duration), and **f** Light dark activity box (dark chamber entries). One-way ANOVA followed by Tukey's post hoc test was used. * Significantly differs from the control group; #significantly differs from the blank group; x significantly differs from Sor-Susp at p < 0.05

As depicted in Fig. 7, the open field test revealed a significant (P < 0.05) reduction in motor activity in mice treated with Sor susp. This was evident from the decrease in crossing squares and rearing frequency compared to the control group. Conversely, mice treated with Sor Nano-Em displayed a notable increase in general motor activity, characterized by a marked rise in crossing squares and rearing frequency compared to Sor susp-treated mice. A non-significant (P > 0.05) difference between the control and blank groups indicated that the observed behavioral changes were primarily due to Sor administration.

In the dark–light activity box test, mice treated with Sor susp exhibited a marked reduction in the frequency of entering the light chamber. They spent less time there, accompanied by a significant (P < 0.05) increase in the frequency of entering the dark chamber and more time spent within it than the control group. Conversely, Sor Nano-Em administration was associated with increased frequency of entering and time spent in the light chamber. Moreover, there was a decrease in the frequency of entering the dark chamber and reduced time spent in it compared to mice treated with Sor susp.

#### Depressive like behavior

**Fig. 8:**
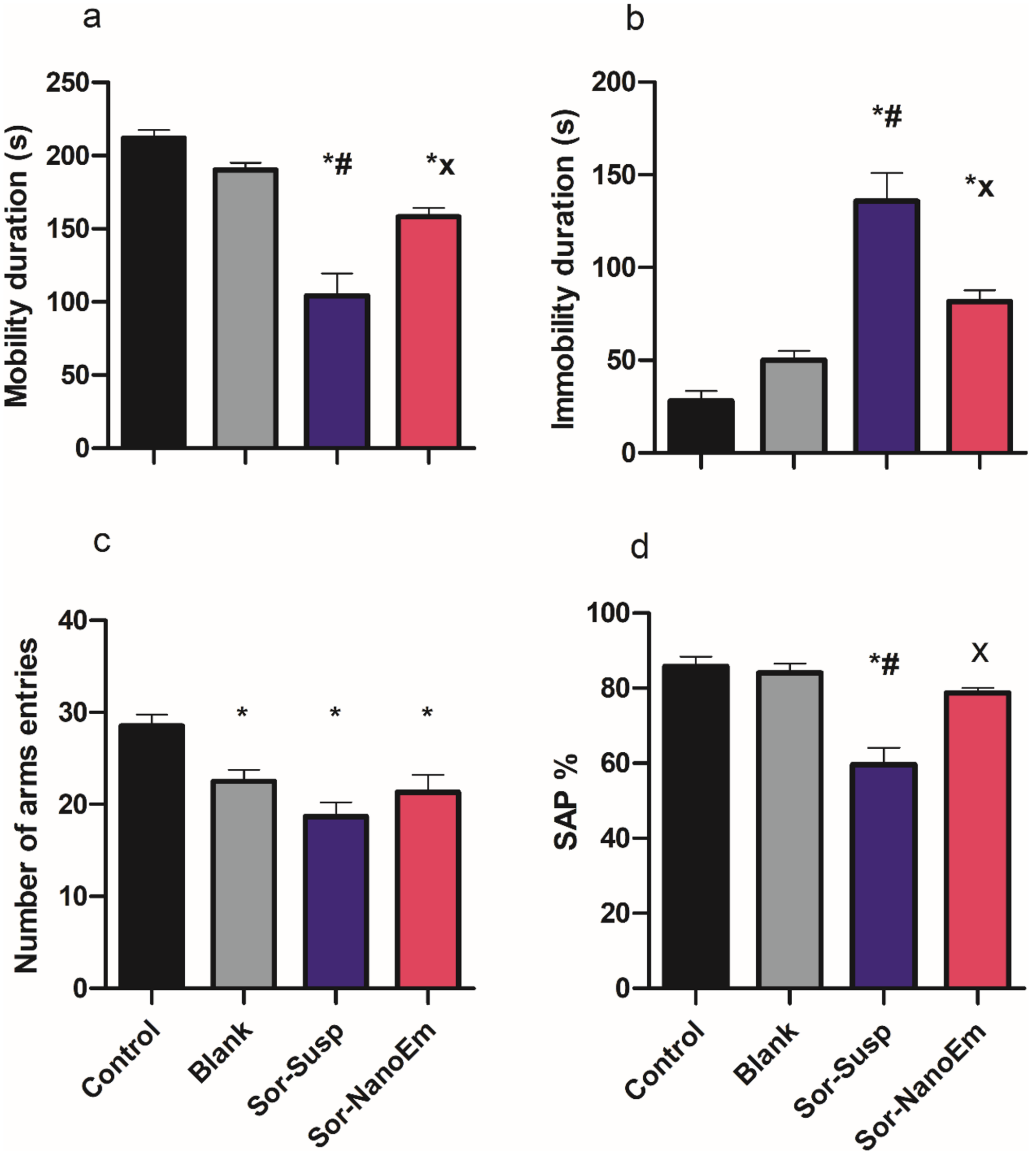
Effect of Sora- Susp and Sor Nano-Em administration on the mice's depressive-like behavior and spatial working memory. **a** Forced swim test (Mobility duration), **b** Forced swim test (Immobility), **c** Y-maze (number of arm entries), and **d** Y-maze (Spontaneous alternation percentage). One-way ANOVA followed by Tukey's post hoc test was used. * Significantly differs from the control group; #significantly differs from the blank group; x significantly differs from Sor-Susp at p < 0.05

As illustrated in Fig. 8a and b, the forced swim test indicated that mice treated with Sor Susp showed a considerable decrease in mobility duration and a notable increase in immobility duration compared to control mice. In contrast, mice treated with Sor NanoEm exhibited a significant (P < 0.05) increase in mobility duration coupled with a marked reduction in immobility duration compared to those treated with Sor Susp.

#### Effect on the Cognitive deficits

The results from the Y-maze test (Fig. 8c and d) indicated that mice treated with Sor Susp exhibited a reduction in the number of arm entries, accompanied by a significant (P < 0.05) decrease in the SAP compared to the control mice. Conversely, mice treated with Sor Nano-Em displayed a considerable increase in arm entries, corresponding to a significant (P < 0.05) rise in the SAP compared to those treated with Sor Susp.

#### Effect on levels of liver enzymes

**Fig. 9:**
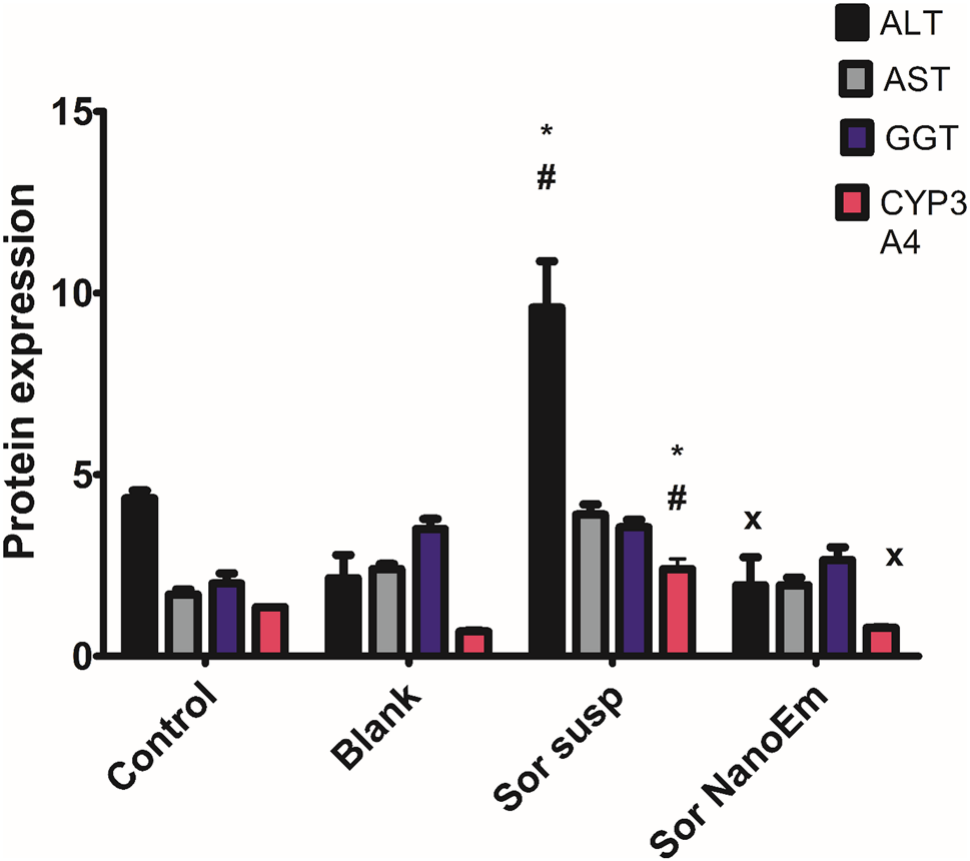
Effects of administering sorafenib suspension in 2.5% CMC (Sor-Susp) or Sor-NanoEm on serum levels of the enzymes: alanine transaminase (ALT, ng/ml), aspartate transaminase (AST, ng/ml), Gamma-glutamyl transferase (GGT, ng/ml) and liver tissue levels of cytochrome P3A4 (CYP3A4, ng/mg protein) using one-way ANOVA followed by Tukey's post hoc test. * Significantly differs from the control group; #significantly differs from the blank group; x significantly differs from Sor-Susp at p < 0.05

Figure 9 illustrates the liver enzyme levels across the various tested groups. The serum level of ALT in mice treated with Sor Susp displayed a significant (P < 0.05) increase, recording the highest value among all groups. Conversely, mice treated with Sor Nano-Em showed normal levels of all evaluated enzymes. Additionally, the measurement of cytochrome P450 3A4 levels in liver tissue revealed non-significant (P > 0.05) differences among most groups, except for mice treated with Sor Susp, which displayed a marked increase in CYP 3A4 levels in liver samples (Fig. 9).

#### Effect on liver tissue levels of Bax and Bcl2

**Fig. 10:**
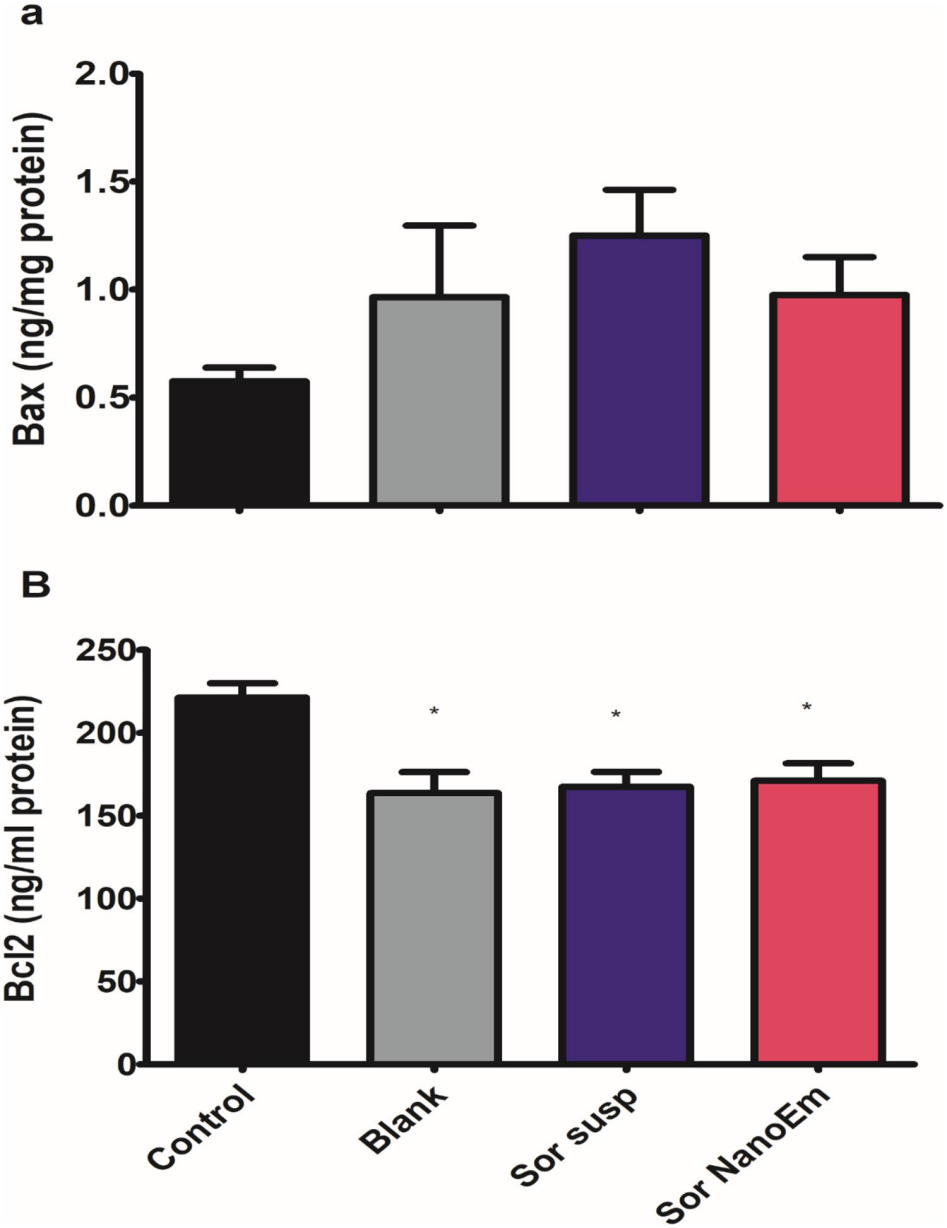
Effects of administering sorafenib suspension in 2.5% CMC (Sor-Susp) or Sor-NanoEm on liver tissue levels of the apoptosis biomarkers; A: Bax (ng/mg protein) and B: Bcl2 (ng/mg protein) using one-way ANOVA followed by Tukey's post hoc test. * Significantly differs from the control group at p < 0.05

The data indicated that the group treated with Sor Susp displayed the highest level of Bax protein. However, this level did not significantly (P > 0.05) differ from the levels observed in other groups (Fig. 10a). Conversely. In contrast, the Bcl2 levels among animals treated with blank, Sor Susp, and Sor NanoEm were not significantly (P > 0.05) different; they were significantly lower (P < 0.05) than the Bcl2 levels recorded for the control group (Fig. 10b).

### Histopathological examination

#### Light microscopic observations

**Fig. 11:**
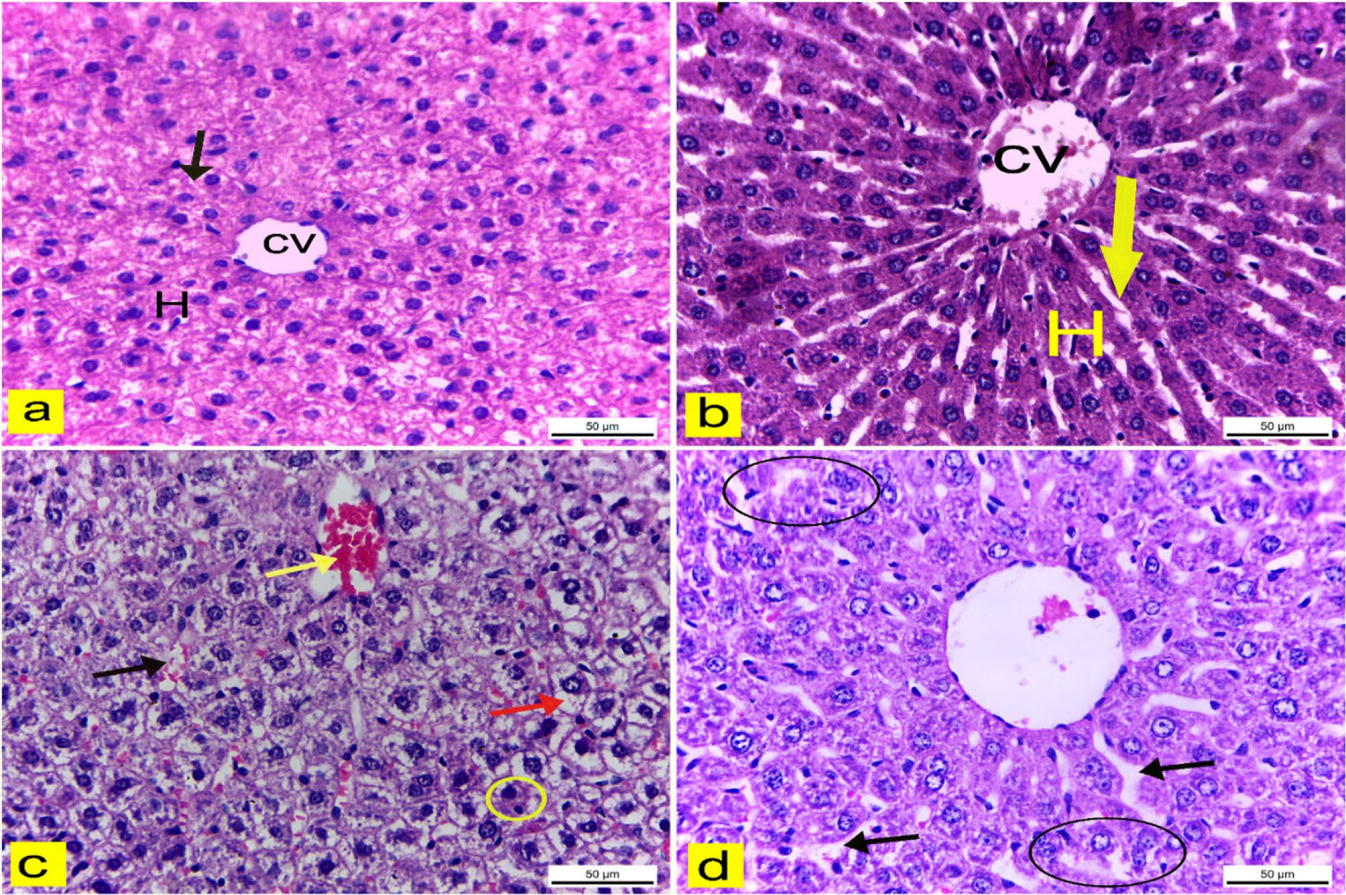
**a**:**d** liver sections of adult male albino mice. H&E X400. **a** & **d** liver of control and blank groups showed regular hepatic cords (H), central vein (CV), and hepatic sinusoids (arrow). **c)** The liver of sorafenib suspension-treated mice revealed a congested central vein (yellow arrow), dilated and congested hepatic sinusoids (black arrow), vacuolar degeneration of hepatocyte cytoplasm (red arrow), and nuclei pyknosis of some hepatocytes (circle).** d** The livers of sorafenib nano-emulsion-treated mice showed disorganization of hepatic cords (circle) and dilation of hepatic sinusoids (arrow)

Histological examination of liver tissues from the control and blank groups displayed a regular arrangement of hepatic cords radiating from a central vein and separated by hepatic sinusoids (Fig. 11a & b). In contrast, liver sections of mice treated with Sor-Susp exhibited abnormalities such as dilated and congested hepatic sinusoids, a congested central vein, cytoplasmic vacuolar degeneration in hepatocytes, and pyknotic nuclei in some hepatic cells compared to the control (Fig. 11c). However, the livers of mice treated with Sor Nano-Em showed fewer histo-architectural alterations, with less disorganization of hepatic cords and fewer dilations of hepatic sinusoids (Fig. 11d).

**Fig. 12:**
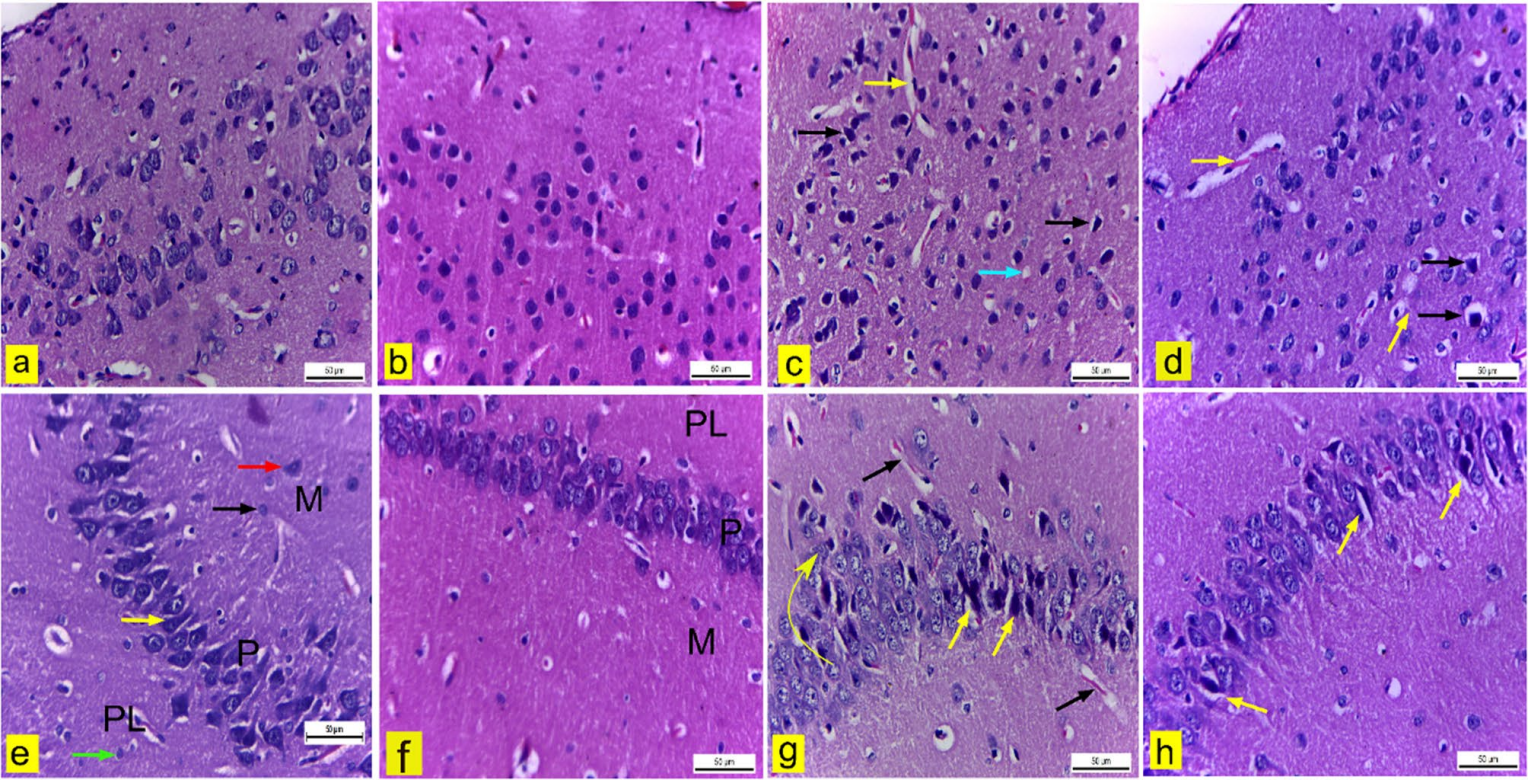
**a: h** Brain tissue of adult male mice. H&E X400. **a & b** cerebral cortex of control and blank mice showed standard structure and distribution of neurons, neuroglia, and neuropil. **c** The cerebral cortex of sorafenib suspension-treated mice revealed congested blood capillaries with perivascular spaces (yellow arrow), pyknotic pyramidal neurons (black arrow), and neuropil vacuolation (blue arrow). **d** The cerebral cortex of mice treated with sorafenib nano-emulsion showed vascular congestion (yellow arrow) and pyknotic pyramidal cells with pericellular space (black arrow). **e & f** hippocampus of control and blank mice exhibited standard structure of the molecular layer (M) composed of neurons (red arrow) and neuroglia (black arrow), the pyramidal cell layer (P) that formed of triangular neurons with vesicular and spherical neurons (yellow arrow) and the polymorphic layer (PL) that consisted of neurons and neuroglia (green arrow). **g** hippocampus of sorafenib suspension-treated mice revealed congested blood capillaries with perivascular space (black arrow), disarrangement of pyramidal cell layer neurons (arched arrow), some pyramidal cells appeared pyknotic with perivascular spaces (yellow arrows). **h** The hippocampus of mice treated with sorafenib nano-emulsion showed few pyknotic pyramidal cells with perineural space (yellow arrow)

H&E-stained sections of the cerebral cortex from the control and blank groups revealed a typical structure and distribution of neurons, neuroglia, and neuropil (Fig. 12a & b). In contrast, the cerebral cortex of mice treated with Sor-Susp showed neuropil vacuolation, congested blood capillaries with perivascular space, and pyknotic pyramidal neurons with pericellular space (Fig. 12c). However, in the cerebral cortex of mice treated with Sor Nano-Em, only a few degenerated pyramidal neurons with pericellular space and vascular congestion were observed compared to those treated with Sor susp (Fig. 12d).

Both the hippocampus sections of the control and blank groups showed standard architecture of the three layers: molecular, pyramidal, and polymorphic. These layers consisted of neurons, neuroglia, and blood capillaries (Fig. 12e & f). However, hippocampal sections from mice treated with Sor-Susp exhibited congested blood capillaries with perivascular spaces in the molecular and polymorphic layers and disarrangement of pyramidal cells in the principal cell layer, including some pyknotic neurons with pericellular spaces (Fig. 12g). In contrast, the hippocampus of mice treated with Sor Nano-Em showed only a few pyknotic pyramidal neurons with pericellular space compared to the Sor-Susp group (Fig. 12h).

#### Immunohistochemical observations

**Fig. 13:**
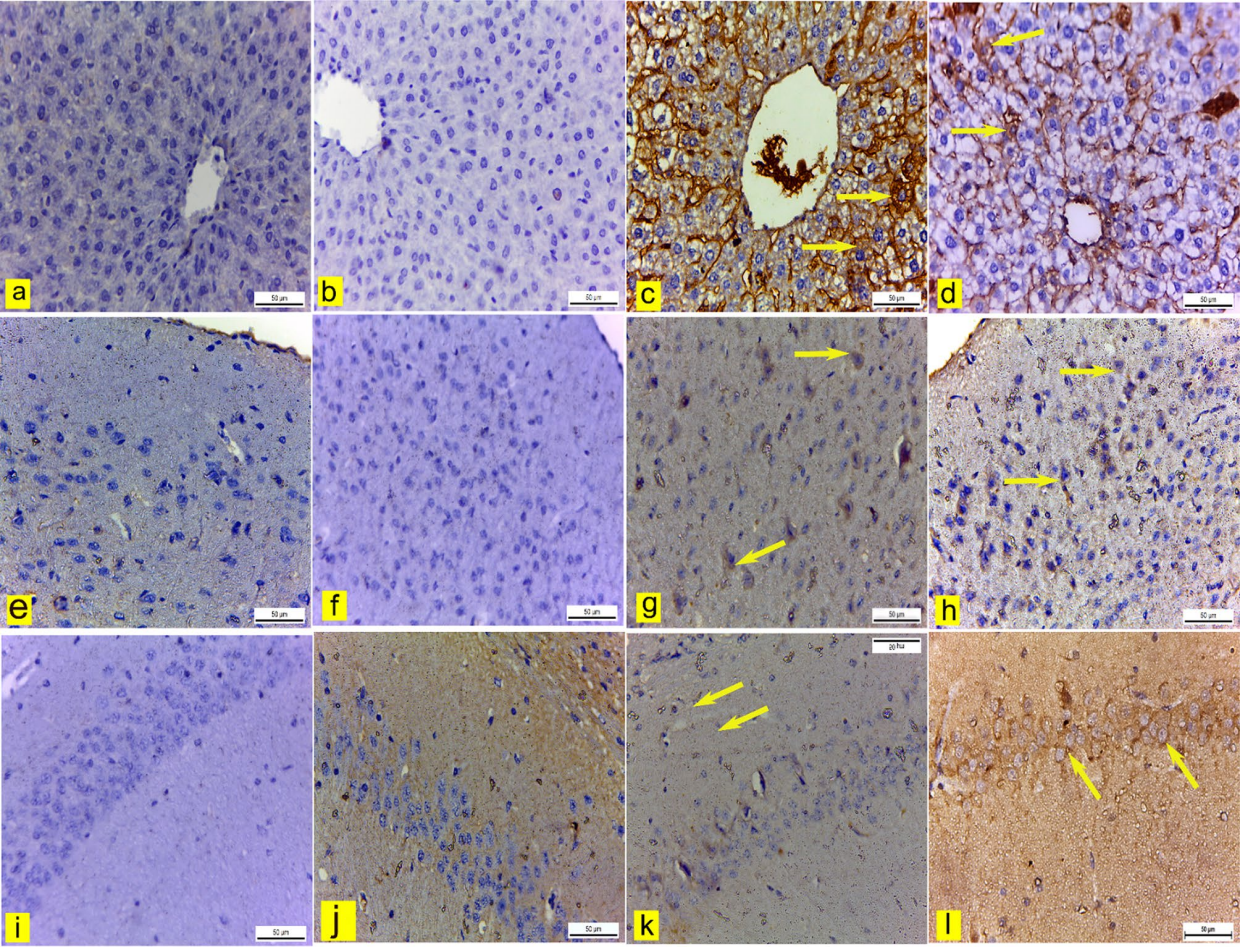
**a: l** Immunohistochemically COX-2-stained liver, cerebral cortex, and hippocampus sections.X400. **a, e, i** control mice and **b, f, j** blank mice liver, cerebral cortex, and hippocampus showed negative COX-2 immunoexpression. **c, g, k** liver, cerebral cortex, and hippocampus of sorafenib suspension-treated mice revealed positive COX-2 immunoexpression in the cytoplasm of some hepatocytes and neurons (yellow arrows). **d, h, l** liver, cerebral cortex, and hippocampus, respectively, of sorafenib nano-emulsion showed mild COX2 immunoexpression

**Fig. 14:**
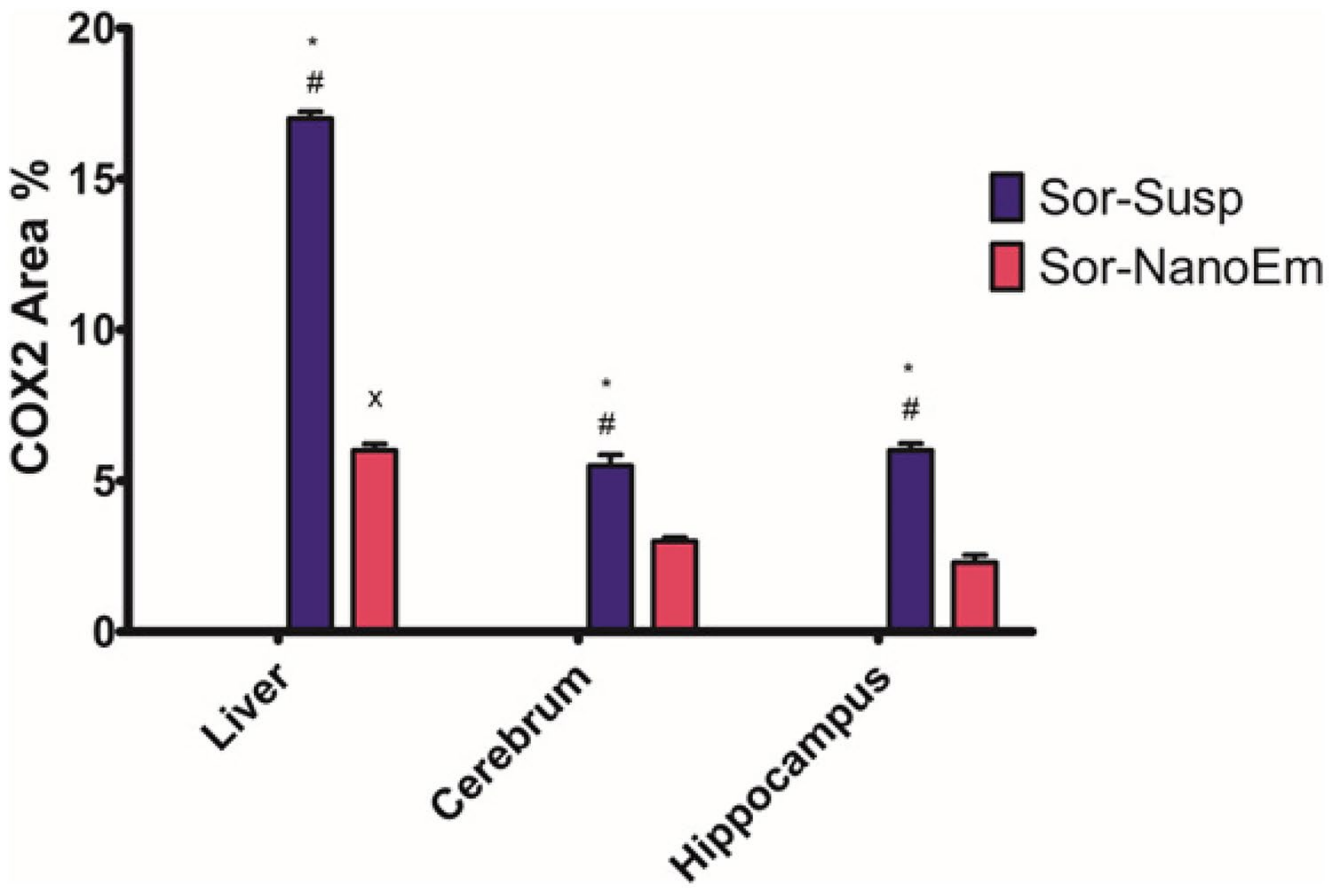
A bar graph showing the effect of sorafenib powder suspended in 2.5% CMC (Sor-Susp) and sorafenib nano-emulsion (Sor-NanoEm) on the area% covered by COX-2 positive immunoreactive cells within the liver, cerebral cortex, and hippocampus of mice. Data are presented as mean values ± SD. One-way ANOVA is followed by Tukey's post hoc test * Significant from the control groups, # Significant from the blank group. **x** Significant from Sor-Susp group, p < 0.05

Immunohistochemical examination of COX-2 in the liver, cerebral cortex, and hippocampus of the control (Fig. 13a, e, & i) and blank (Fig. 13b, f, & j) groups displayed negative cytoplasmic COX-2 immune-expression. However, liver tissue, cerebral cortex, and hippocampus of mice treated with Sor-Susp revealed significant positive cytoplasmic COX-2 immunoreactivity in some hepatocytes and neurons (Fig. 13c, g, & k and Fig. 14). Conversely, mice treated with Sor Nano-Em displayed a significant reduction in COX-2 immuno-expression in the liver and a non-significant reduction in neurons of the cerebral cortex and hippocampus compared to the Sor-Susp group (Fig. 13d, h, & I and Fig. 14).

**Fig.15:**
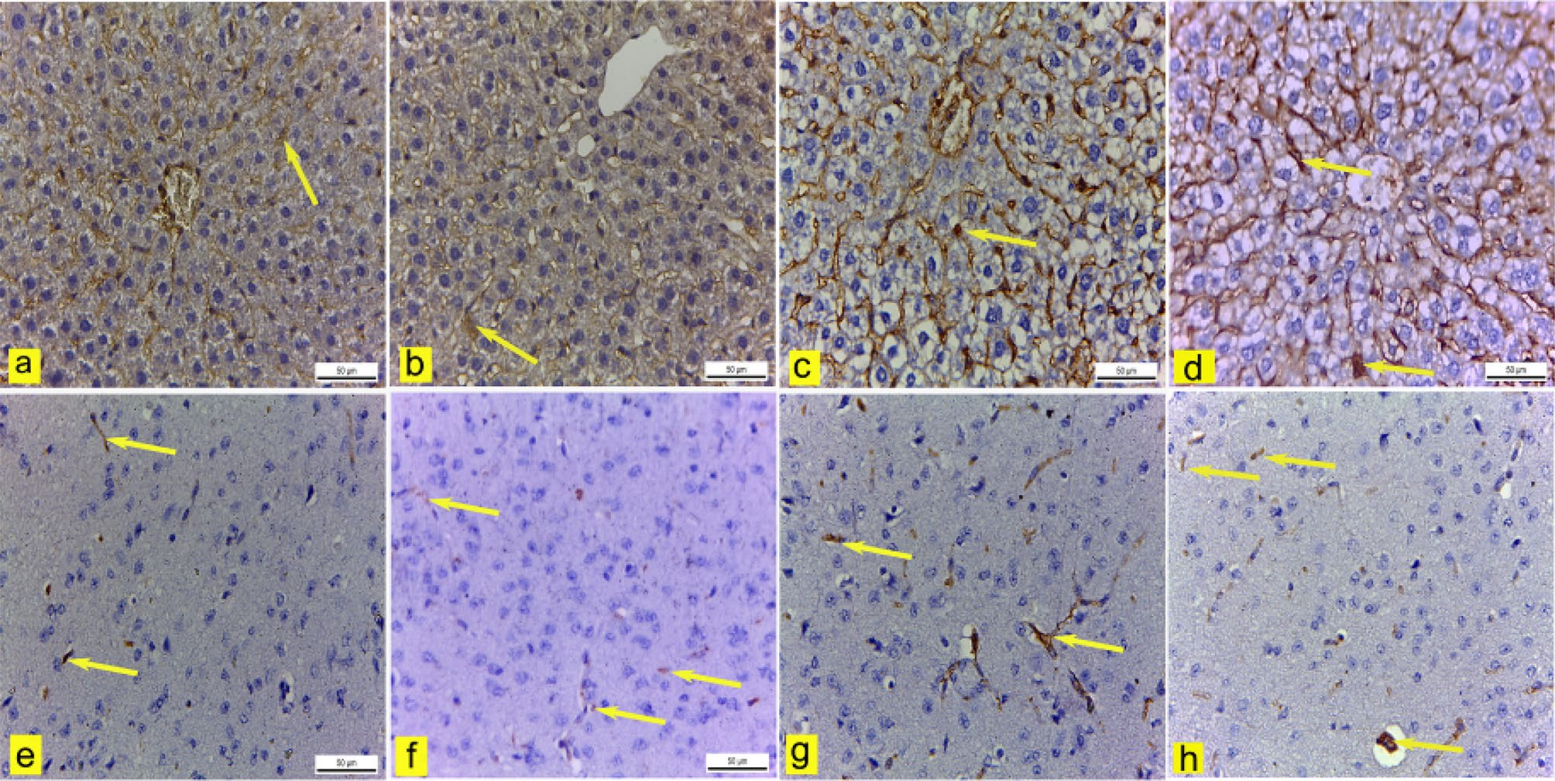
**a: h** Immunohistochemically CD31-stained liver and cerebral cortex sections.X400. **a & b** liver of control and blank mice showed mild CD31 immunoexpression of sinusoidal endothelial cells (yellow arrow). **c** The liver of mice treated with sorafenib suspension showed moderate CD31 immunoexpression (yellow arrow). **d** The liver of mice treated with sorafenib nano-emulsion had mild CD31 immunostaining (yellow arrow). **e & f** Control and blank mice's cerebral cortex blood capillaries had mild CD31 immunopositivity (yellow arrow). **g** The cerebral cortex blood capillaries of mice treated with sorafenib suspension showed moderate CD31 immunoreactivity (yellow arrow). **h** The cerebral cortex blood capillaries of mice treated with sorafenib nano-emulsions showed mild CD31 immunostaining (yellow arrow)

**Fig. 16:**
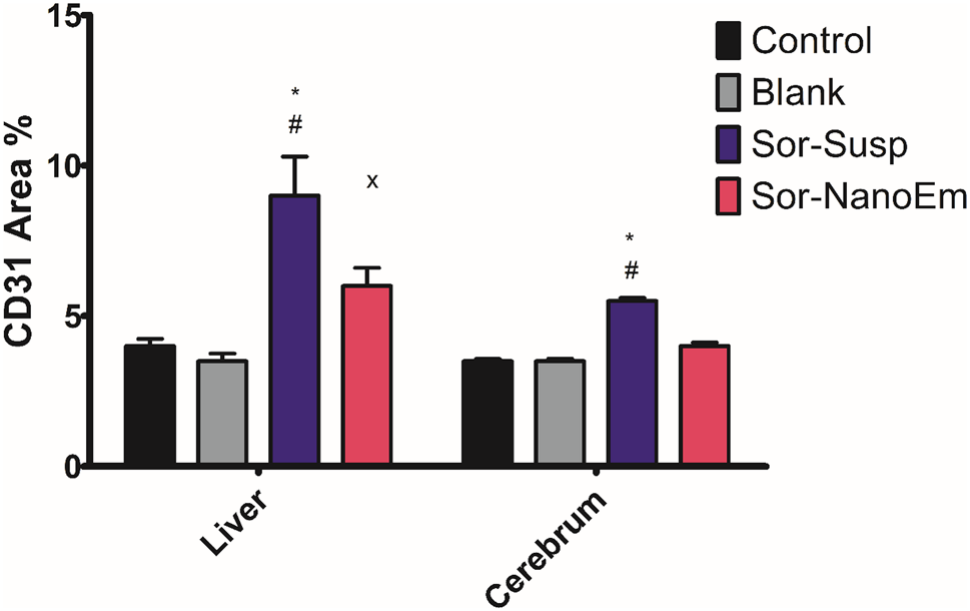
A bar graph showing the effect of sorafenib powder, suspended in 2.5% CMC (Sor-Susp) and sorafenib nano-emulsion (Sor-NanoEm) on the area % covered by CD31 positive immunoreactive cells within the liver and cerebral cortex of experimental mice. Data are presented as mean values ± SD. One-way ANOVA is followed by Tukey's post hoc test * Significant from the control groups, # Significant from the blank group. **x** Significant from Sor-Susp group, p < 0.05

In the liver of control and blank mice, there was mild CD31 immunopositivity of sinusoidal endothelial cells (Fig. 15a & b and Fig. 16). Conversely, the liver of mice treated with Sor-Susp revealed a significant increase in CD31 immunoexpression compared to control and blank mice (Fig. 15c and Fig. 16). However, sinusoidal endothelial cells showed a significant reduction in CD31 immunoreactivity in mice treated with Sor-NanoEm compared to Sor-Susp (Fig. 15d and Fig. 16).

Regarding the cerebral cortex blood capillaries of control and blank mice, there was mild CD31 immunopositivity (Fig. 15e & f and Fig. 16). In contrast, cerebral cortex blood capillaries expressed significantly elevated CD31 immunoreactivity in mice treated with Sor-Susp (Fig. 15g and Fig. 16). However, cerebral cortex blood capillaries of mice treated with Sor-NanoEm showed a non-significant reduction in CD31 immunoreaction compared to Sor-Susp (Fig. 15h and Fig. 16).

## Discussion

Sor is considered a first-line medicinal agent to manage HCC. However, it has many drawbacks, as described previously. The current study aimed at encapsulating Sor into nano-emulsion followed by in vitro and in vivo comparative studies with Sor susp to tackle any improvement of its anticancer activity or safety due to its encapsulation into nano-emulsion.

The formed nano-emulsion was characterized for particle size zeta potential, and TEM determined its morphology. The particle size of Sor NanoEm was significantly (P < 0.05) higher than the size of blank nano-emulsion, which might be due to drug encapsulation into the nano- droplet. This is consistent with the literature, where Ifosfamide, a chemotherapeutic agent encapsulated into Nano-Em, showed a larger droplet size than the blank Nano-Em [51].

PDI value was ≤ 0.3. This indicated that the formed Nano-Em is monodisperse with a lower tendency of accumulation and, therefore, could be suitable for drug delivery application [51, 52, 52, 53]. Although the low negativity of zeta potential was recorded, the Sor NanoEm was stable with a low tendency of agglomeration as indicated by PDI value and TEM images; the low negativity might be attributed to Tween 80, a non-ionic surfactant, which was used as an emulsifying agent for preparation of nano-emulsion. Stability of nano-emulsion could be explained by the steric repulsive forces of Tween 80 (non-ionic surfactant) that are able to overcome the attractive van der Waals forces and therefore, be able to stabilize nano-emulsion against agglomeration [23, 54–56]

TEM images showed nano-droplets with a spherical shape with no signs of aggregation; however, the droplet size was slightly smaller than the size recorded by Malvern Instrument, and this might be due to different techniques applied, and this is consistent with the literature [50, 51, 57]. Both pH and viscosity values are acceptable for oral application.

Sor release from Sor NanoEm was tracked by UV–Vis spectrophotometric analysis. The analysis method was validated by determining the accuracy and precision of inter-day and intra-day variations. The determined values of precision indicated a high degree of repeatability and reproducibility. Following AOAC guidelines, the following equations, % RSD of repeatability = C ^−0.15^ and % RSD of repeatability = 2 C ^−0.15^, were used to calculate the theoretical RSD% for intra-day and inter-day variations, respectively, where C is the analyte concentration expressed as mass fraction. A valid method's accepted practical %RSD ranged between ½ and 2 times the theoretically calculated values. The obtained values matched with what was recommended by AOAC guidelines, assuring the validity of the UV–visible spectrophotometric analysis method used in the current Study.

Concerning the release study, Sor was ultimately released from its solution form after 8h, which is consistent with the literature [58]. This is contrary to a lower release percentage recorded for Sor NanoEm simultaneously, followed by a sustained release behavior after 10 h (zero-order release kinetics). The fast release of Sor from Sor Nano-Em over the first 10 h might be explained by the large surface area of the nano-droplet (the droplet size was 121.75 ± 12). This is consistent with a previous study [59], where linezolid, an antitubercular drug encapsulated into Nano-Em, showed zero-order release kinetics.

Concerning the cytotoxicity study, Sor-NanoEm significantly (P < 0.05) improved Sor's antiproliferative and cytotoxic effects on HepG2 cells. HepG2 cells were inhibited more efficiently with Sor Nano-Em than Sor-Susp, indicating that the nano-emulsion formula is more effective against HepG2 cells than Sor susp. Consequently, the required dose of Sor is reduced with Nano-Em. This might lead to fewer side effects and increase its efficacy, making Sor a safer and more effective chemotherapeutic drug for HCC treatment [60].

To ensure the safety of the new formula, a mouse-based LD 50 assay was carried out. First, the LD50 values of Sor Nano-Em administrated orally were estimated in mice according to procedures described previously [39]. Symptoms such as severe diarrhea, paralysis, and seizures were observed in the intoxicated mice. Different doses of Sor Nano-Em were selected to analyze further changes in mice after intoxication quantitatively. It was found that the toxic effect of Sor Nano-Em in mice was dose-dependent. Massive diarrhea and fluid loss in mice before death indicated that dehydration could be one of the leading causes of the death of mice from Sor Nano-Em overdose. Accordingly, 30 mg/kg was determined as a safe therapeutic dose to be applied in vivo.

Upon investigating the influence of the Nano-Em on Sor pharmacokinetics, a comparative study was conducted versus Sor susp. The nano-emulsion significantly (P < 0.05) increased the following pharmacokinetics parameters, including Cmax, AUC, and t_1/2_ of Sor, along with reducing its elimination, clearance, and volume of distribution. This can ensure better drug characteristics with favorable prolonged action, fewer frequency of administration, and, therefore, lower adverse events.

Chemotherapeutic medications are associated with toxic adverse effects, including sickness behavior [60, 61]. The underlying mechanisms of these adverse effects have yet to be fully elucidated. In the present study, we investigated some of the toxic effects of Sor therapy and the ability of the new NanoEm formula to overcome these deleterious effects on the brain and livers of experimental mice through direct action or via lowering the therapeutic effective dose needed. Sickness behaviors, including anxiety-like behavior, depressive-like behavior, and cognitive deficits, were investigated in addition to several biochemical and immunohistopathological analyses. Sor-Susp treated mice showed a decrease in motor activity, a highly anxious state portrayed by an increase in the dark chamber duration and frequency associated with a reduction in the light chamber duration and frequency. Also, they displayed a depressive-like state evidenced by a decrease in the mobility duration and an increase in the immobility duration. These behavioral changes were associated with a cognitive deficit represented by a reduced SAP compared to control. These findings are in agreement with previous studies [42, 62–65] that investigated the sickness behavior associated with chemotherapy administration, including doxorubicin, cyclophosphamide, and cisplatin. However, the administration of Sor Nano-Em was able to reverse all these changes compared to Sor-Susp. Growing evidence suggests that several potential medications, including Sor, displayed enhanced absorption and solubility after encapsulation into NPs [66]. Bearing in mind that the capability of the brain to take only up to 5% of the initial NP dose, with rapid clearance from the circulation and a decrease in the blood–brain barrier crossing [67]. This could be the reason for the reduction of sickness behavior associated with Sor susp administration.

Histopathologically, the brain tissue of mice treated with Sor suspension displayed degenerative changes in the cerebrum, hippocampus, and cerebellum histo-architecture. This may be attributed to the passage of Sor through the blood–brain barrier. On the other hand, treatment with Sor nano-emulsion resulted in less degenerative changes. Immunohistochemically, Sor susp treatment caused inflammation and angiogenesis in the neuronal tissue of mice. These results indicated several side effects associated with the medicinal application of Sor susp that might negatively affect or limit its therapeutic application. Taken together, fewer side effects recorded with the application of Sor NanoEm on the brain revealed the merits of Sor encapsulation into nano-formulations.

Assessing the levels of hepatic enzymes is a common way to determine the state of liver function. These enzymes leak into the bloodstream when hepatocytes are damaged, and as a result, they serve as indicators of liver damage. Increased levels of these biochemical variables could trigger carcinogenesis as they frequently correlate with the prevalence of hepatotoxicity (Awad et al., 2023). In this study, the elevated levels of ALT in the Sor-Susp group represented the progression of liver damage. The findings showed that Sor Nano-Em administration improved liver function more than Sor-Susp therapy, demonstrating the nano-emulsion formula's capacity to restore serum enzyme levels in hepatotoxic conditions by reducing liver damage and boosting anti-cancer activity. Our results are consistent with recent research where scientists confirmed their agents' hepatoprotective and anti-cancer properties by restoring normal levels of liver enzymes [68–70]. Our results revealed increased alterations of cytochrome P450 in Sor-Susp group samples, dramatically impacting metabolism, therapeutic efficacy, and adverse drug reactions for itself and other drugs. Furthermore, elevated CYP levels can induce liver injury through toxic metabolites and reactive oxygen species production [71]. The Sor-NanoEm restored normal levels of CYP, preventing the deleterious effect found in the suspended formula.

In the current study, the histopathological investigations revealed alterations in the hepatic tissue structure of mice treated with Sor suspension, including dilated and congested hepatic sinusoids with loss of hepatocyte standard structure. These findings agreed with Alkhatib and colleagues, who reported that mice treated with Sor revealed alterations in hepatic tissue and dilated sinusoids [72]. Strumberg attributed the hepatotoxic effect of Sor to its oxidative metabolism in the liver [73]. Immunohistochemical examination revealed severe inflammatory lesions in the hepatic tissue of mice exposed to Sor suspension, evidenced by significant intense COX2 expression. These results align with the findings of Ting and colleagues, who stated that rats injected with Sor showed severe inflammatory changes in hepatic tissue by H&E stain [74].

Moreover, angiogenesis was observed in mice treated with Sor suspension, evidenced by a significant increase in CD31 immunoexpression in endothelial cells of hepatic sinusoids compared to control mice. Otherwise, mice treated with Sor-NanoEm revealed a substantially reduced angiogenesis via decreased CD31 expression compared to the Sor-suspension-treated group.

Conversely, mice treated with Sor Nano-Em revealed fewer degenerative, inflammatory, and angiogenesis changes in hepatic tissue than those treated with Sor suspension.

## Conclusion

The superior performance of the Sor-NanoEm formulation in vitro, where it successfully suppressed HepG2 cell viability and induced apoptosis, demonstrated increased anti-cancer efficacy. Additionally, Sor Nano-Em showed enhanced pharmacokinetic characteristics and reduced Sor dose with a lower incidence of side effects when administrated in vivo. In vivo studies indicated that mice given Sor Nano-Em improved their behavioral profile and recovery of essential biomarkers, such as COX-2 inflammatory markers and liver enzymes. Notably, the structural integrity of the liver and brain returned to normal.

These results highlighted that encapsulation of Sor into nano-emulsion might be a potential alternative strategy for its application for better therapeutic management of carcinogenic patients compared to conventional Sor therapy.

## Data Availability

Data will be made available from the corresponding author upon request.

## Abbreviations

ALT: Alanine Transaminase
AST: Aspartate Aminotransferase
Bax: Bcl2 associated X protein
Bcl2: B-cell leukemia/lymphoma 2
CD31: Cluster of differentiation 31
COX-2: Cyclooxygenase 2 (COX-2)
CYP3A4: Cytochrome P450 3A4
GGT: Gamma-Glutamyl Transferase
HCC: Hepatocellular Carcinoma
LD50: Lethal dose 50
SAP: Spontaneous Alternation Percentage
Sor: Sorafenib
NanoEm: Nano-Emulsion
Susp: Suspension
